# Structure and Dynamics of Cas9 HNH Domain Catalytic State

**DOI:** 10.1038/s41598-017-17578-6

**Published:** 2017-12-08

**Authors:** Zhicheng Zuo, Jin Liu

**Affiliations:** Department of Pharmaceutical Sciences, University of North Texas System College of Pharmacy, University of North Texas Health Science Center, Fort Worth, TX 76107 United States

## Abstract

The bacterial CRISPR-Cas9 immune system has been harnessed as a powerful and versatile genome-editing tool and holds immense promise for future therapeutic applications. Despite recent advances in understanding Cas9 structures and its functional mechanism, little is known about the catalytic state of the Cas9 HNH nuclease domain, and identifying how the divalent metal ions affect the HNH domain conformational transition remains elusive. A deeper understanding of Cas9 activation and its cleavage mechanism can enable further optimization of Cas9-based genome-editing specificity and efficiency. Using two distinct molecular dynamics simulation techniques, we have obtained a cross-validated catalytically active state of Cas9 HNH domain primed for cutting the target DNA strand. Moreover, herein we demonstrate the essential roles of the catalytic Mg^2+^ for the active state formation and stability. Importantly, we suggest that the derived catalytic conformation of the HNH domain can be exploited for rational engineering of Cas9 variants with enhanced specificity.

## Introduction

The **c**lustered **r**egularly **i**nterspaced **s**hort **p**alindromic **r**epeats (CRISPR)/CRISPR-associated protein 9 (spCas9) system from *Streptococcus pyogenes* has been repurposed as a powerful and versatile genome-editing tool and used in various living cells and organisms, demonstrating an enormous potential for future therapeutic applications^1–4^. Guided by a chimeric single-guide RNA (sgRNA), the endonuclease Cas9 generates site-specific breaks in the double-stranded DNA (dsDNA) target^5,6^. Recognition and cleavage of dsDNA strictly requires the presence of a protospacer adjacent motif (PAM) in the non-target DNA strand (ntDNA) and depends on the base-pair complementarity of the target DNA strand (tDNA) to the RNA guide template^5,6^. spCas9 adopts an overall bilobed architecture, in which the sgRNA:tDNA heteroduplex resides within a central channel between the α-helical recognition (REC) and nuclease (NUC) lobes, while the displaced ntDNA threads into a side channel within the NUC lobe (Supplementary Fig. 1a)^7–10^. The NUC lobe comprises two metal-ion-dependent nuclease domains, termed as HNH and RuvC, which are responsible for cleaving the tDNA (via a one-metal-ion mechanism^11,12^) and ntDNA (via a two-metal-ion mechanism^11–13^), respectively.

To crystalize nuclease/substrate complexes, the cleavage reaction has to be prevented by artificial means, e.g., mutation of catalytic residues and use of transition-state analogs or divalent ions that do not support catalysis. These strategies inevitably perturb the active center architecture and lead to alterations in the number and location of metal ions^13^. It is thus not surprising that none of the spCas9 crystal structures in different binding forms that have been solved over the past few years captures a fully active state for either the RuvC or HNH domains^7–10,14^. Sternberg *et al*.^15^ reported that these conformations observed in existing crystal structures are the inactive intermediates along the conformational activation pathway. The crystal structures of other Cas9 homologs, such as those from *Francisella novicida* (FnCas9) and *Staphylococcus aureus* (SaCas9)^16,17^, also exhibit the HNH nuclease domains in the inactive conformations. In our previous work, we used molecular dynamics simulations and identified the catalytically competent state of the RuvC domain primed for cleaving the ntDNA^18^. However, we were unable to capture the catalytic conformation of the HNH domain required for cleaving the tDNA in that study^18^. In contrast with the RuvC domain, the active center of the HNH domain is surprisingly distant from the scissile phosphate on the tDNA in all available structures^7–10^, with a separation ranging from ~14 Å in the complete DNA duplex bound pre-catalytic state (Supplementary Figs. 1a,b) to ~46 Å in the RNA-only bound inactive state. More recently, Palermo *et al*. delineated the free energy landscape underlying Cas9 conformational activation by the enhanced dynamics methods^19^, though the activated conformation needs further validation. Thus, obtaining a reliable catalytic state of the Cas9 HNH domain has been of special focus to both experimental biologists and the computational biophysicists, as such a structure can provide one important missing link in understanding Cas9 binding, activation, and cleavage mechanisms and guides structure-based Cas9 engineering with enhanced specificity^20,21^. In addition, two of the most recent single-molecule Förster resonance energy transfer (smFRET) studies suggested that divalent metal ions are necessary for Cas9 conformational activation toward catalysis^22,23^. Still, understanding how the metal ions aid the transition of the HNH domain to the catalytic state remains elusive.

The knowledge of the structure and dynamics of the catalytic state of the HNH domain is critical for improvement of Cas9 specificity. Off-target effects pose a major challenge for Cas9-mediated genome-editing applications requiring a high level of precision^24^; therefore, much effort is needed to increase the fidelity of CRISPR-Cas9 with regard to the generation of off-target mutations, especially in the clinical setting^25^. Recently, two publications proposed that Cas9-guide RNA possesses more energy than necessary for optimal recognition of its intended target sequence, which leads to cleavage at mismatched off-target sites^20,21^. Based on the inactive structure of the Cas9-sgRNA complex with a partial dsDNA target^10^, several high-fidelity Cas9 variants have been designed and validated for the elimination of off-target effects, demonstrating that the structure-guided Cas9 engineering is a robust strategy for specificity improvement^20,21^. Given that all of the previous efforts were based on an inactive structure, structural information regarding other Cas9 conformational states, especially the catalytic state, could enable further optimization of the CRISPR-Cas9 genome-editing tool.

Molecular dynamics (MD) is a powerful computer simulation method and has been proven to be especially useful for elucidating the structure-function relationships of biological macromolecules. With two distinct MD simulation techniques, herein we show a cross-validated catalytically active state of the Cas9 HNH nuclease domain that had yet to be experimentally characterized using structural techniques. We note that the reliability of this catalytic model is supported by various experiments^5,6,9,13,14,20,22,23,26,27^. Meanwhile, we also demonstrate the essential roles of Mg^2+^ for the formation and stability of the catalytic state. Most importantly, the derived catalytic model provides novel, valuable structural information that can be exploited for rational engineering of more high-fidelity Cas9 variants.

## Results

### The HNH Domain Samples Larger Conformational Space in the Absence of ntDNA

In obtaining the HNH domain active state from the inactive state structure using molecular dynamics simulations, the biggest challenge is sampling adequate conformational space in a reasonably short time-scale. From our initial MD simulations and structural observation^7,18,28^, we suspected that the ntDNA might impose spatial constraints on the conformational dynamics of the HNH domain in the pre-catalytic state (Supplementary Figs. 1a,b). In other words, if we break the established interactions with the ntDNA, the HNH domain could theoretically sample larger conformational space due to relief of constraints. Even though the increased flexibility could lead to a higher possibility for the HNH domain to move further away from the scissile phosphate of tDNA, as suggested in a recent computational study^28^, it may also increase the probability of the HNH domain to access or move closer to the catalytic state in a shorter time-scale that is suitable for MD simulations. To test whether removal of ntDNA leads to larger conformational sampling space for the HNH domain, we performed three groups of long time-scale conventional MD (cMD) simulations starting from the pre-catalytic structure^7^, in which the ntDNA was removed (**G1** and **G2**, Table 1) or retained (**G10**, Table 1). Meanwhile, the accelerated MD (aMD) method^29,30^ was implemented to enhance the sampling of the system without ntDNA at two different boost levels (**G3** and **G4**, Table 1). The cumulative effective sampling time was up to 14.3 µs, including 11 µs of cMD and 3.3 µs of aMD.

**Table 1:** Summary of MD simulations for Cas9 complex systems without non-target DNA strand (w/o ntDNA) and with ntDNA.

	Group	Simulation method^§^	Starting structure	Production time per run [ns]	No. of runs	Mg^2+^ present at HNH domain?
w/o ntDNA	**G1**	cMD	Crystal structure	2500	2	
	**G2**		(PDB code: 5F9R)	1000	1	√
	**G3**	aMD^**Ed**^	Extracted from G1	650	2	
	**G4**	aMD^**dual**^		1000	2	
	**G5**	tMD	Extracted from G1/G2	100	2	
	**G6**	cMD	Extracted from G5	800	2	√
	**G7**	cMD	Extracted from G6	800	2	
	**G8**.**1**	cMD^**ens**^	Extracted from G1/G2	500	10	√
	**G8**.**2**		Extracted from G8.1		10	√
	**G8**.**3**		Extracted from G8.2		10	√
	**G8**.**4**		Extracted from G8.3		10	√
	**G9** ^*^	cMD	Extracted from G6/ G8.4	850	2	√
with ntDNA	**G10**	cMD	Crystal structure	1000	2	
			(PDB code: 5F9R)	1500	2	

^**§**^cMD, conventional unbiased MD; aMD^**Ed**^, accelerated MD with dihedral boot only; aMD^**dual**^, accelerated

MD with simultaneous dihedral and total potential boost; tMD, targeted MD; cMD^**ens**^, ensemble cMD.

^*^Asp839Ala mutant simulated.

**Figure 1:**
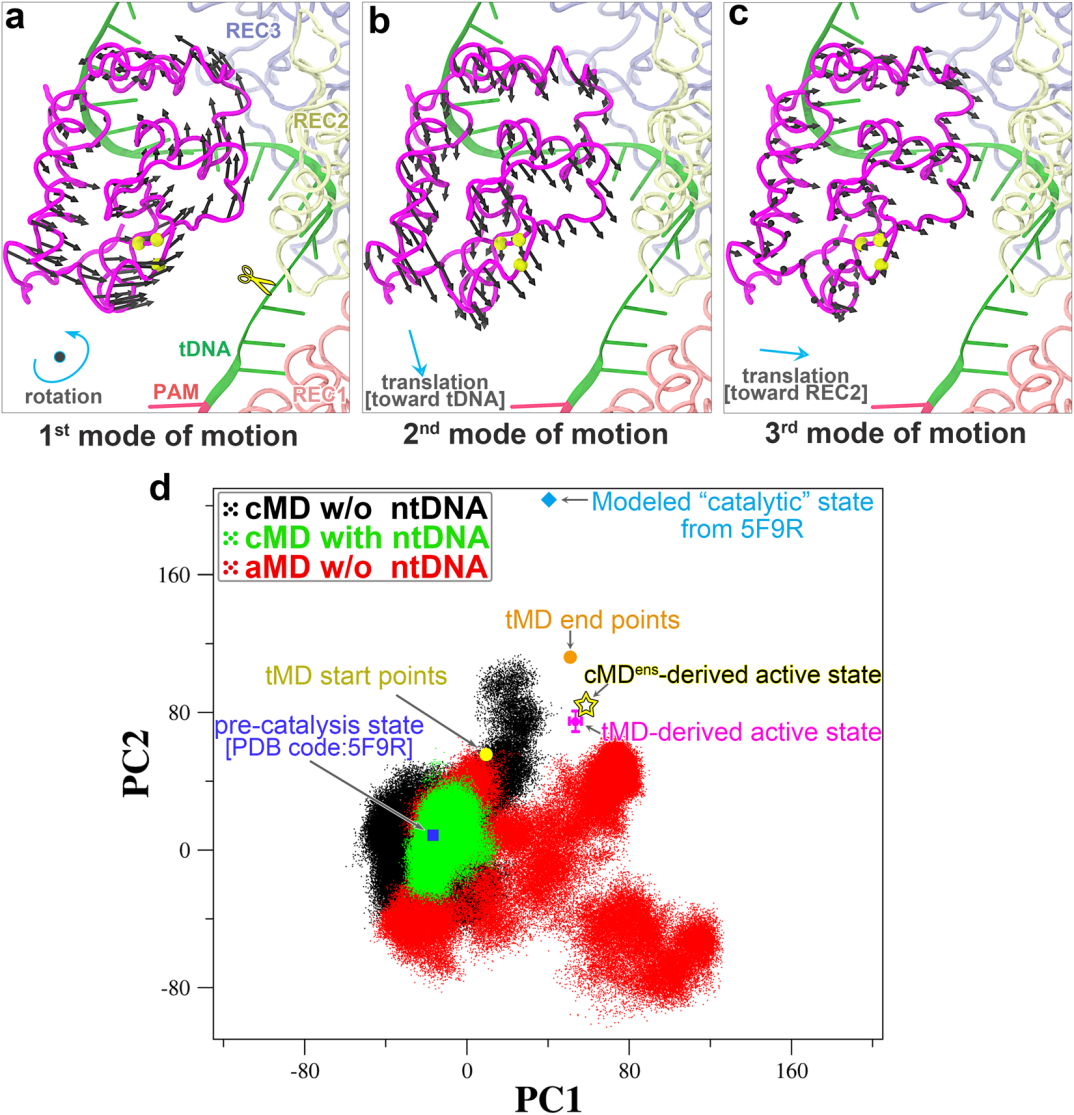
Cas9 HNH domain motions and conformational flexibility characterized by principal component analysis. (**a**–**c**) Visualization of the top three dominant motions for the HNH domain. The first motional mode depicts a rotation motion around an axis perpendicular to the plane, while the second and third modes describe a translational movement toward the tDNA and REC2 domain, respectively. The Cα atoms of the three HNH catalytic residues are represented as yellow van der Walls spheres. (**d**) Overlap of the projections of the conventional MD simulations without ntDNA (cMD w/o ntDNA, black dots), conventional MD simulations with ntDNA (cMD with ntDNA, green dots) and accelerated MD simulations without ntDNA (aMD w/o ntDNA, red dots) onto the first two eigenvectors calculated from the whole trajectories for the HNH domain. The pre-catalytic state (PDB code: 5F9R; highlighted as a magnified square filled in blue), its modeled “catalytic” state (as a filled cyan diamond) from the crystal structure of T4 Endo VII complex with a DNA substrate (Supplementary Text), and the start and end points (shown as yellow and orange filled circles, respectively) for the targeted MD (tMD) simulations were also projected onto the subspace defined by the first two PCA modes, along with the targeted MD (tMD)- and ensemble conventional MD (cMD^ens^)-derived catalytic states (an average over 100 data points is reported; represented as a red cross and a yellow pentagon outlined in black, respectively). All the trajectories were best-fitted to the Cas9 protein (excluding the HNH domain) of the pre-catalytic crystal structure (PDB code: 5F9R), and the coordinate covariance matrix was computed over the HNH domain for subsequent analysis (see details in Supplementary Text).

To compare the conformational spaces sampled with the two different systems and by the two different simulation approaches, we first performed a principal component analysis (PCA) to determine the dominant motions of the HNH domain (Supplementary Text). PCA is a multivariate statistical technique applied to systematically reduce the number of dimensions needed to describe protein essential dynamics^31,32^. The first three PCA modes, accounting for 70% (37% + 23% + 10%) of the overall motion, revealed a rotational motion along an axis perpendicular to the central channel between the two Cas9 lobes (Fig. 1a), and translational movements toward the tDNA (Fig. 1b) and the REC2 domain (Fig. 1c), respectively. Apparently, a combination of these dominant motions towards the REC lobe and tDNA would lead the HNH domain toward the cleavage site on the tDNA. Subsequently, we projected individual sets of simulation trajectories onto the subspace defined by these three PCA vectors (Supplementary Fig. 2a–c). As we expected, the accessible conformational space of the HNH domain in the ntDNA-bound system was approximately a subset of those in the ntDNA-free system (Fig. 1d and Supplementary Fig. 2). Moreover, we calculated the distances of Ser867 (on the HNH domain) to Ser355 (on the REC1 domain) and to Asn1054 (on the RuvC domain) that were first selected for labeling in a bulk FRET experiment^15^ and later in the smFRET experments^22,23^, and obtained similar results to the PCA (Supplementary Fig. 3). In our dsDNA-bound model, the ntDNA 5′-end cleavage product was not included, and thus the sampling space is likely to be further confined in the context of full-length ntDNA due to interactions between the 5′-end stretch and the HNH domain^7,18,28^.

Compared to cMD, aMD explored much broader conformational space, especially along the first principal component (Fig. 1d and Supplementary Fig. 2), which depicts a rotation motion of the HNH domain (Fig. 1a). However, the third motional mode is more prominent in cMD than in aMD (Supplementary Fig. 2f), suggesting that the HNH domain displays a larger-scale translation toward the REC lobe in cMD (Fig. 1c). This observation indicates that the enhanced dynamics, aMD, does not always lead to further progression along certain transition pathways compared to the conventional MD method. Interestingly, as indicated below, the third motional mode favors the conformational activation of the HNH domain.

To this end, we demonstrated that the HNH domain samples larger conformational space in the absence of ntDNA, and cMD is more appropriate in searching for the HNH domain active state as aMD brings appreciable internal structural distortion (Supplementary Fig. 4 and Supplementary Table 1). As the microsecond time-scale samplings with cMD and aMD were unable to obtain an HNH conformation in sufficiently close proximity to the cleavage site on tDNA for catalysis, in the following sections, we will present two different strategies to capture the converged catalytically active state of the HNH domain.

### Targeted-MD Revealed the Catalytically Active State of the HNH domain

**Figure 2:**
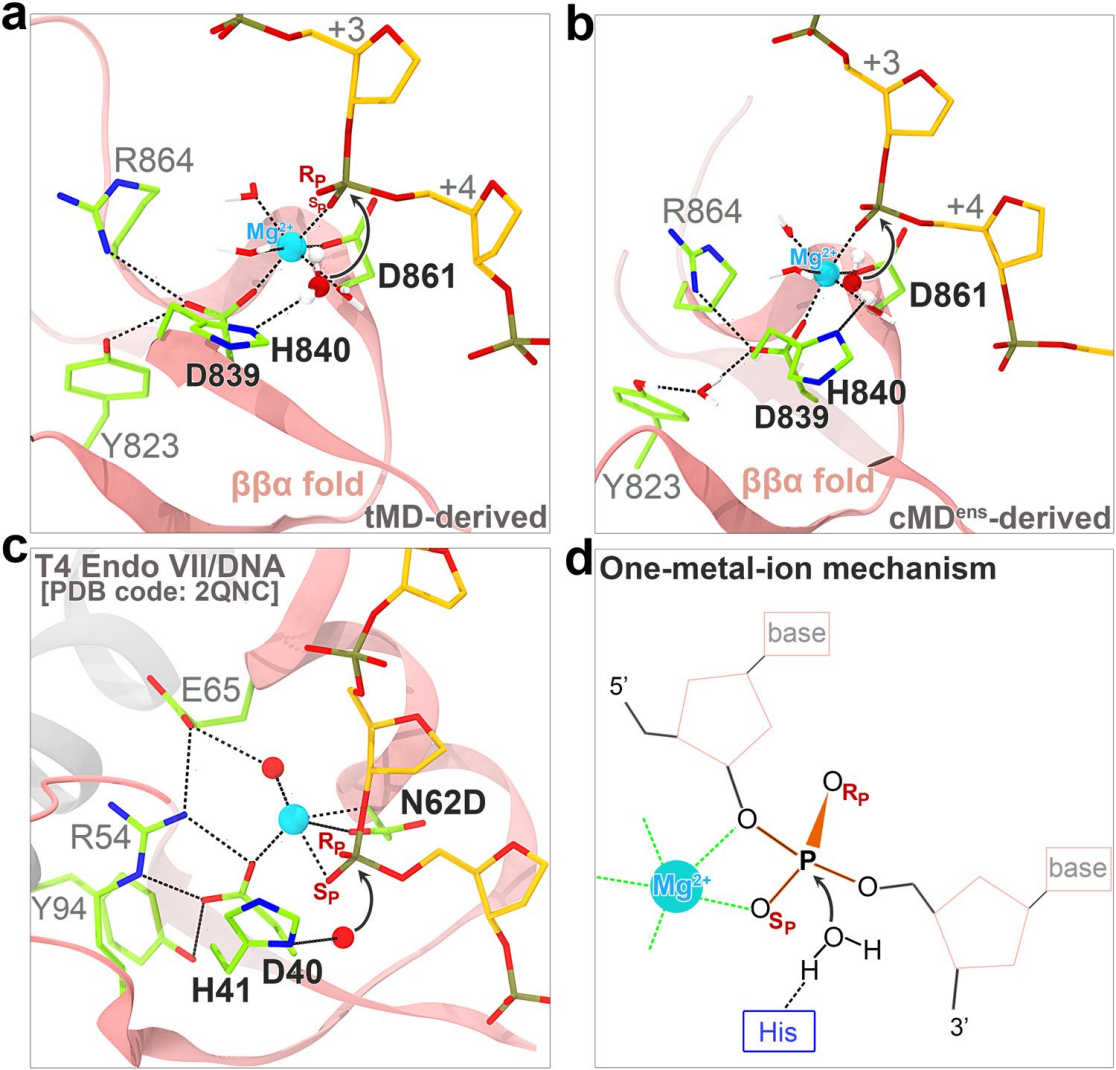
Catalytic state coordination at the interface of HNH ββα fold and tDNA (**a**,**b**) and comparison with the one-metal-ion catalysis by T4 Endo VII (**c**). (**a**,**b**) The representative coordination configurations derived from post targeted MD (tMD) simulations (**a**) and conventional ensemble MD (cMD^ens^) simulations (**b**) through cluster analysis (see Supplementary Text). (**c**) Close-up view of the active center of T4 Endo VII (N62D) resolving a DNA Holliday junction (PDB code: 2QNC). (**d**) Schematic representation of the one-metal-ion dependent catalysis in ββα-metal nucleases. The pro-Sp and pro-Rp oxygens of the scissile phosphate are indicated with Sp and Rp, respectively. The putative active residues in Cas9 HNH domain and the corresponding residues in T4 Endo VII are labeled in boldface. In panels a to c, the nucleobases are omitted for clarity, and the potential nucleophilic water are depicted in a ball and stick model and colored by atom types (O, red; H, white). Mg^2+^ is shown as a cyan sphere and the nucleophile is denoted by a curved arrow. The dashed lines indicate the coordination bonds involving Mg^2+^ and hydrogen-bonds.

One of the strategies we used was a targeted MD (tMD) simulation^33,34^, which is an approach that enables conformational transition between two known states by application of external forces. First, we selected the homologous T4 Endonuclease VII (Endo VII) complex with a DNA Holliday junction^26^ as the template upon which to build the target conformation of the HNH domain, which is the putative “active” conformation model (Supplementary Text and Supplementary Fig. 5). Instead of a single static target, we built multiple targets based on each snapshot structure from the above sets of long cMD simulations. We selected the snapshot structures with a minimum root-mean-square deviation (RMSD) (~10 Å) from its own “target” as the starting point of the tMD (Supplementary Text). Obviously, the modeled “target” conformation does not represent the actual “active” state and varies depending on which snapshot is employed as the initial structure. With a small force constant and a low RMSD decreasing rate, we carried out the tMD simulations and observed the expected conformational transition of the HNH domain, largely due to its intrinsic global flexibility as well as its internal structural rigidity (Supplementary Fig. 4 and Supplementary Table 1). In the framework of one-metal-ion mechanism (Fig. 2d)^11,12^, one Mg^2+^ was then introduced at the reaction interface between the HNH domain and the tDNA. After performing thorough post tMD simulations using conventional MD (**G6**, Table 1), we obtained a reasonable catalytically active conformation.

The Mg^2+^ at the catalytic center formed a favorable octahedral coordination with six surrounding oxygen atoms from different species (Fig. 2a). In addition to the three water molecules, the residues Asp839 and Asp861 on the ββα motif and the scissile phosphate (pro-Sp oxygen involved) between the nucleotides +**3** and +**4** of tDNA each contributes a coordination ligand (Fig. 2a). The above observation is consistent with the per-residue energy decomposition data afforded by the MM-GBSA approach (Supplementary Fig. 6), confirming the important role of the residues in stabilizing the Mg^2+^ cation. In contrast, His840 contributes marginally to Mg^2+^ binding, which is consistent with its major role as the general base that activates the nucleophile. Notably, the His840 side chain participates in a hydrogen-bond with a potential nucleophilic water molecule that is oriented for an in-line attack on the scissile bond. Tyr823 and Arg864 appeared to play an especially important structural role in stabilizing the catalytic Asp839 side chain through hydrogen-bonding interactions. We presumed that such hydrogen-bonds aid in the proper orientation of Asp839 for coordination and catalysis. Indeed, primary sequence analysis shows that the amino acid tyrosine is strictly conserved among different types of CRISPR-Cas9, while the basic amino acid arginine (or lysine) is highly conserved among the Type II-A Cas9 orthologs^14^.

Overall, the three active resides (Asp839, His840 and Asp861) and the two other residues (Tyr823 and Arg864) (Fig. 2a) are spatially and functionally analogous to the corresponding residues [Asp40, His41, Asn62, Tyr94 (on the other subunit) and Arg54] in the T4 Endo VII (Fig. 2c)^26^. Despite the similarities, the Mg^2+^ cation in this case was not positioned as near to the leaving group 3′-O as in the Endo VII system^26^, which was also observed at the reaction interface between the Cas9 RuvC domain and ntDNA in our prior study with the same force fields^18^. Apart from the potential issue with the Mg^2+^ parameters, this deviation might be partially related to the subtle differences between the two enzymes beyond the coordination center. In Endo VII, for instance, there exists an additional acidic residue (Glu65) that hydrogen-bonds to a coordinating water molecule above the bound Mg^2+^ (Fig. 2c). Furthermore, a recent simulation work reported a distance of 4~6 Å between His840 and the scissile phosphate^19^, which is comparable to that of ~5.5 Å observed here (Supplementary Fig. 7). In summary, the coordination composition and geometry captured here closely match those present in the T4 Endo VII/DNA complex, indicating the formation of a catalytically active state of the Cas9 HNH domain.

### Robustness of the tMD-derived Catalytic State is Confirmed by Unbiased Ensemble MD Simulations

**Figure 3:**
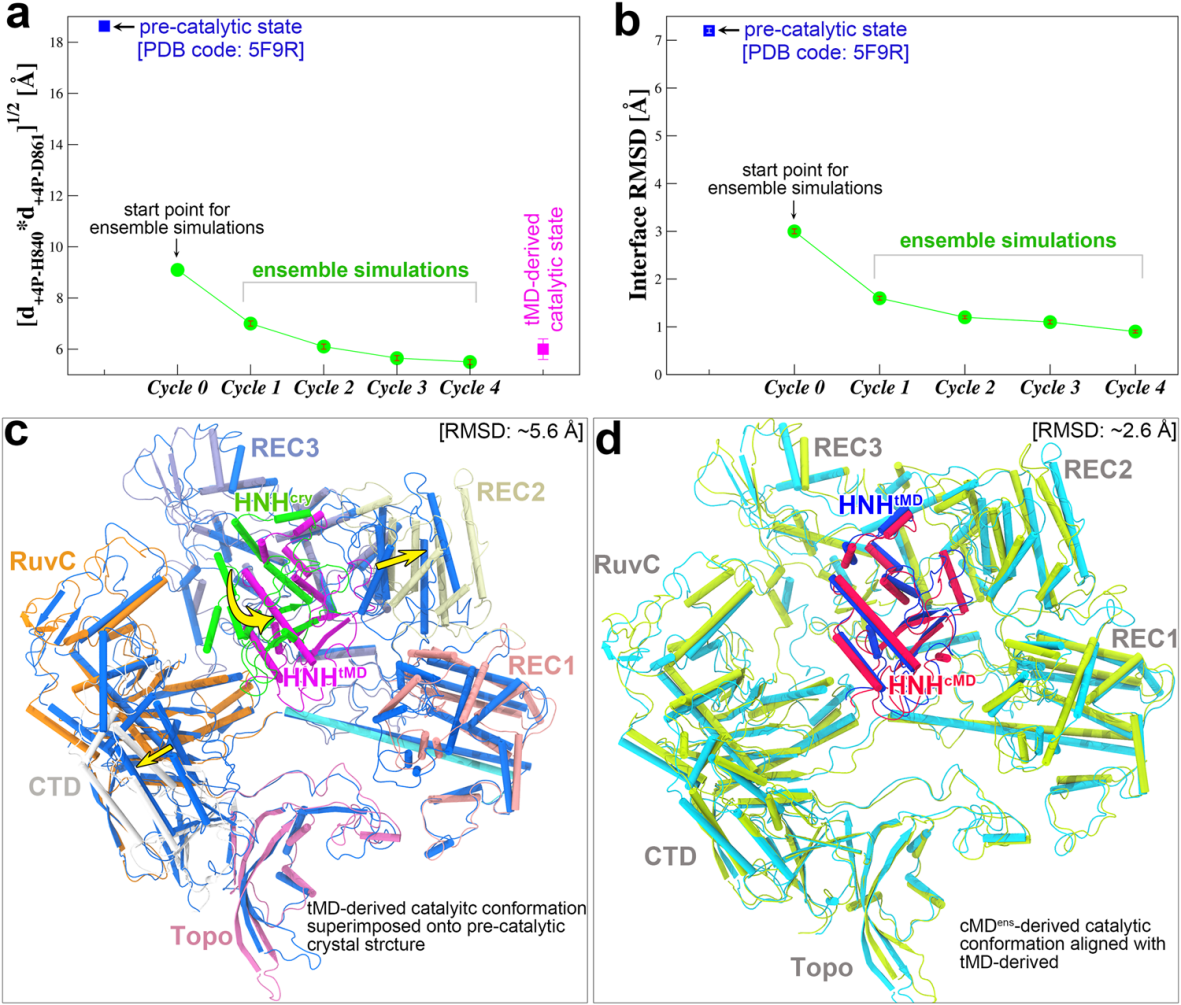
Comparison of the targeted MD (tMD)-derived and ensemble conventional MD (cMD^ens^)-derived catalytic Cas9 conformations and Comparison with the crystal structure in pre-catalytic state. (**a**) Variation of the minimum geometric mean of the distances of +4**P** to His840 (**d**_**+4P-H840**_) and to Asp861 (**d**_**+4P-D861**_) as a function of the cycle number in the course of the ensemble simulations. (**b**) Variation of minimum binding interface RMSDs from the tMD-derived catalytic state as the cycle number increases. An average over fifty data points is reported in a and b. (**c**) Structural superposition between the tMD-derived catalytic state and crystal pre-catalytic state (PDB code: 5F9R). The HNH domain within 5F9R (labeled as HNH^cry^) is highlighted in green and the remainder of Cas9 colored blue, with the largest domain movement (involving HNH, REC2 and CTD) denoted by a yellow arrow. The tMD-derived catalytic structure is colored by domains: HNH (labeled as HNH^tMD^), magenta; REC1, pink; REC2, pale yellow; REC3, iceblue; RuvC; orange; CTD; grey; Topo, hotpink, BH, cyan. See also Supplementary Fig. 8 for the result with cMD^ens^. (**d**) Structural alignment between the tMD- and cMD^ens^-derived catalytic Cas9 conformations. tMD- and cMD^ens^-derived catalytic HNH domain (respectively labeled as HNH^tMD^ and HNH^cMD^) is respectively highlighted in blue and red, with the remainder of Cas9 colored cyan and yellow-green in the respective structures. The bound nucleic acids are omitted for clarity.

The above tMD-based strategy used to capture the catalytic state in essence is based on a modeled putative “target” state. Although we treated the building process with special considerations, the impact of potential artificial effects on the tMD-derived catalytic model cannot be definitively ruled out. Therefore, we performed a series of conventional MD ensemble simulations (cMD^ens^) starting from the original pre-catalytic crystal structure (PDB code: 5F9R) to determine if we could reach the same catalytic state using the unbiased MD approach. We developed a method called “Step-by-step MD”. The basic idea behind this method is to extract from a set of MD simulations the structure that most resembles the active state as the new starting point for a new set of the simulations. Step-by-step, we can efficiently sample the desired conformational space without any artificial forces. As the actual catalytic state is not known, it is challenging to choose the structure that most resembles the catalytic state. Here, we used the geometric mean of the distances of + 4**P** (the scissile phosphate) to two catalytic residues His840 and Asp861 as a metric to monitor the conformational transition of the HNH domain. It is assumed that the smaller this value is, the closer the conformation is to the target active conformation. From the sets of long cMD trajectories (**G1** and **G2**, Table 1), we extracted a structure bearing a minimum value of ~9 Å as the starting point for the ensemble simulations (Fig. 3a). In this starting point, one Mg^2+^ was located at the reaction center. In each cycle, the ensemble simulations were seeded from a structure snapshot from a previous cycle bearing the lowest value of the above geometric mean (Fig. 3a), which represents the core of our sampling approach here. More details regarding the cMD^ens^ are presented in Supplementary Information.

**Figure 4:**
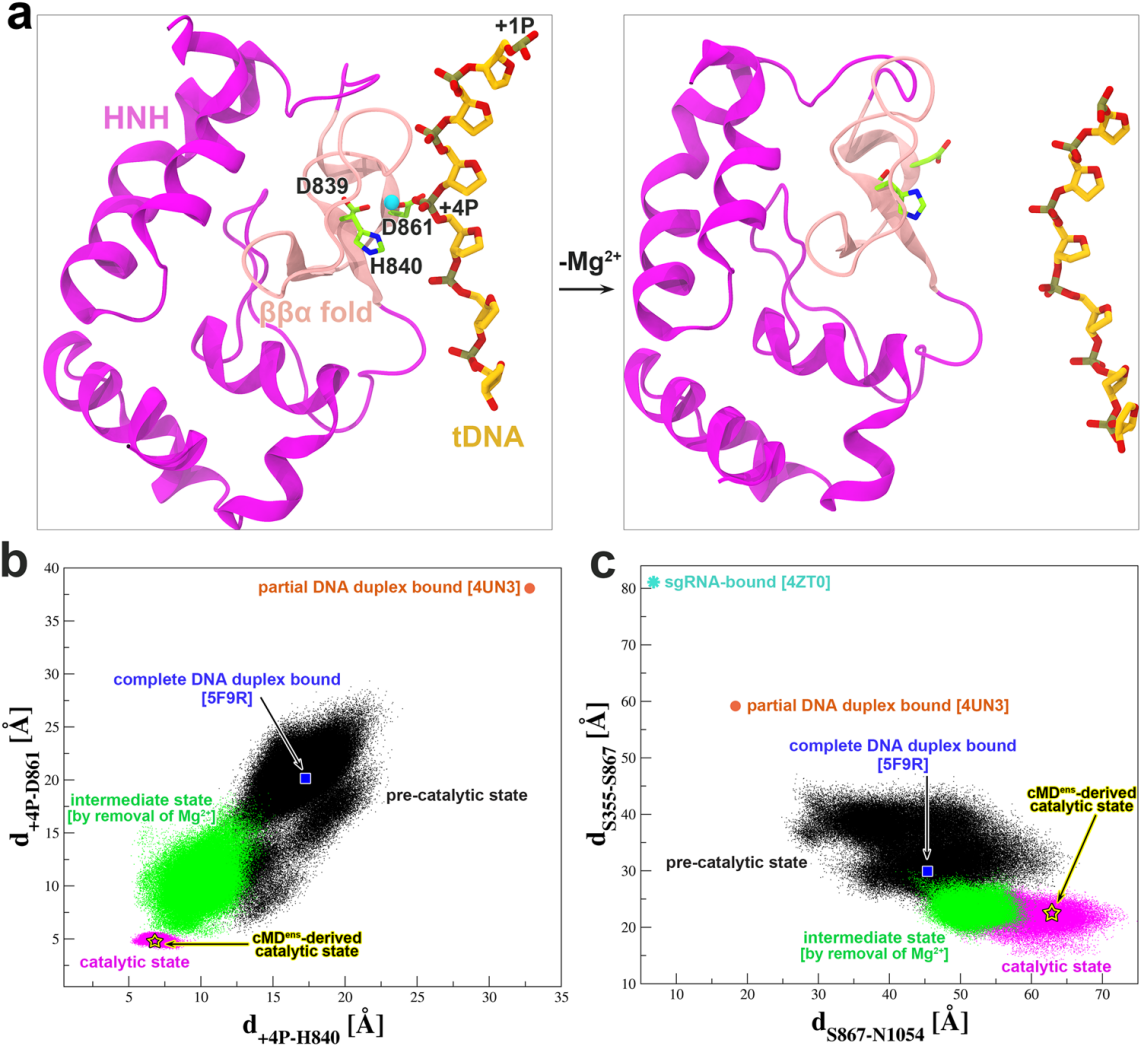
Mg^2+^-aided conformational transition to catalytic state. (**a**) Comparison of the representative HNH conformations from the cMD simulations with Mg^2+^ bound (left) and with Mg^2+^ removed (right) at the reaction interface. The bound Mg^2+^ is shown as a cyan sphere and the HNH active residues are represented in a stick model. (**b**) Scatter plot of the +4**P** distances to His840 (**d**_**+4P-H840**_) and Asp861 (**d**_**+4P-D861**_) calculated from different sets of cMD simulations. The Cγ and P atoms were selected for measurement. (**c**) Scatter plot of the distance pair for Ser867/Asn1054 (**d**_**S867-N1054**_) and Ser355/Ser867 (**d**_**S355-S867**_) from different sets of cMD simulations. The Cα atoms were calculated here. The residue pairs of Ser867/Asn1054 and Ser355/Ser867 were used to characterize the conformational states of HNH domain in previous FRET experiments (Supplementary Fig. 3). If available, the corresponding distance pairs obtained from different Cas9 complex crystal structures are mapped on each plot. Of note, in 4UN3, the loop where Asn1054 resides is disordered and we report an average of the distances calculated from respective modeled structures using 4ZT0, 4ZT9 and 5F9R as a template. The pentagrams indicate the catalytic state derived from the conventional ensemble MD (cMD^ens^) simulations (an average over 100 data points is reported).

Through four cycles (**G8**.**1**–**G8**.**4**, Table 1), the above geometric mean stabilized at ~6 Å (Fig. 3a), which is comparable to that observed for the tMD-derived catalytic state (Fig. 4a,b). Accordingly, the RMSD of the reaction interface from the tMD-derived catalytic state declined from an initial value of ~3 Å to ~1 Å (Fig. 3b). Moreover, the Mg^2+^-involved coordination composition and configuration here (Fig. 2b) are essentially the same as those derived from tMD (Fig. 2a), with the exception that Tyr823 was engaged to Asp839 via an intercalated water molecule, again confirming the structural role of Tyr823 around the reaction center. These observations therefore demonstrated formation of the cMD^ens^-derived catalytic state and confirmed the previously identified cleavage site location of 3 nucleotides from the PAM^5,6^. On the other hand, we performed a set of mutant simulations in which the putative active residue Asp839 was replaced with alanine (**G9**, Table 1). As a result, the HNH domain largely departed from the tDNA, and the general base His840 moved beyond the attacking distance to the scissile phosphate (Supplementary Fig. 7). The mutant simulations thus verify the proposed key role of Asp839 in catalysis^9,14^, providing another piece of evidence that validates our catalytic model. In addition, a most recent smFRET study^27^ estimated a distance decrease of ~5 Å between the fluorophore labeling sites at Ser355Cys and Ser867Cys while Cas9 switching from the cleavage-impaired state (equivalent to the pre-catalytic state here) to the cleavage-competent state. Considering the uncertainty in FRET measurements, this range can be compared to our simulations that give an average decrease of 6.7 Å in the inter-Cα distance of Ser355 and Ser867 after transition to the catalytic conformation (Fig. 4c).

With the active state formation, the Cas9 protein underwent prominent conformational changes, as observed from either of the post tMD and cMD^ens^ simulations. The overall Cα RMSD from the initial crystal structure is near 6 Å, wherein the HNH domain displayed a largest RMSD of ~11 Å as expected, followed by the CTD and REC2 domains with a RMSD around 7~8 Å (Supplementary Table 2). In the absence of ntDNA, the CTD domain moved markedly outward, resulting in wide opening of the side channel within the NUC lobe, which is thereby poised for substrate loading (Fig. 3c and Supplementary Fig. 8). When RMS fitting to themselves, the HNH and REC2 domains exhibited a much smaller RMSD of less than 2 Å (Supplementary Table 2), indicating a concerted motion of REC2 domain with the HNH domain. By contrast, the RMSD of the CTD domain was reduced by a relatively small range of 3.5 Å, suggesting considerable variation in its internal conformation in addition to the above large-scale reorientation. Taken together, the results reveal the highly mobile nature of individual Cas9 domains, consistent with previous experimental and computational studies^7–10,14,28^.

Overall, the two differently derived catalytic conformations were highly superimposable (Fig. 3d). The global RMSD between them is approximately 2.6 Å (Supplementary Table 2), partially contributed by the flexible CTD domain and relative domain movements. In line with these results, the HNH domain assumed a similar orientation and conformational state between the two catalytic states, as characterized by the principal component analysis (Fig. 1d) and the distance pair between the FRET-labeled residues (Fig. 4c). Furthermore, the vast majority of newly formed interactions with the HNH domain are common between the two catalytic conformations (Supplementary Figs. 9–10, and Supplementary Table 3) as mentioned below. In aggregate, all these data suggest a good convergence of the tMD- and cMD^ens^ -derived catalytic models.

### Mg^2+^ is Indispensable for Activation of the Catalytic State

Our previous work with the Cas9 RuvC domain revealed that Mg^2+^ is able to induce the formation of the active state for cleaving the ntDNA^18^. Likewise, beyond its catalytic role, we reason that Mg^2+^ could also facilitate conformational activation of the HNH domain. To test this hypothesis, we removed the coordinated Mg^2+^ from the above catalytic conformation (Fig. 4a) and performed microsecond-level conventional MD simulations (**G7**, Table 1). In the absence of Mg^2+^, we can envision two distinct consequences for the HNH domain, i.e., either departing from the tDNA or staying docked at the tDNA without noticeable reorganization.

We first monitored the changes in the distance pair of +4**P** to His840 (**d**_**+4P-H840**_) and to Asp861 (**d**_**+4P-D861**_) at the cleavage interface (Fig. 4a). Their geometric mean increased from 6.0 Å in the catalytic state simulations to 10.5 Å on average, indicating detachment of the HNH domain from the tDNA. Further comparison with the cMD simulations starting from the pre-catalytic state clearly showed that absence of Mg^2+^ leads the HNH domain to an intermediate state between the catalytic and pre-catalytic state (Fig. 4b). We speculate that with a longer sampling time, the HNH domain would ultimately reach the pre-catalytic state as observed in the crystal structure^7^. A similar trend was also observed with the FRET residue pairs (Ser867/Asn1054 and Ser355/Ser867) (Fig. 4c), yet the states are relatively less distinguishable than was the case with the reaction interfacial residues (Fig. 4b), which is probably due to a longer time needed for remote conformational relaxation. Consequently, the binding free energy of Cas9 to tDNA reduced by ~30 kcal/mol compared to the catalytic state (not including the entropic contribution) (Supplementary Text). More specifically, the non-bonded interaction energy of the HNH ββα motif with the scissile phosphate and flanking nucleotides decreased by ~64 kcal/mol (Supplementary Text). Given the stable Mg^2+^-mediated catalytic conformation, we argue that the HNH domain is least likely to be separated from its opposite cleavage site unless the reaction is over. Taken together, these results evidence that Mg^2+^ is essential for the formation and stability of the Cas9 HNH domain active state, as was also observed for the RuvC domain^18^. Likewise, the findings here are in good accordance with the most recent smFRET experiments^22,23^.

### The Catalytic State Provides New Structural Information for Specificity Enhancement

Accompanying the active state formation, remarkably, the HNH domain established a plenty of new interactions with the REC lobe (including REC1, REC2 and REC3), bridge helix (BH), tDNA and sgRNA, and predominantly involved the charged and polar residues (Supplementary Figs. 9-10 and Supplementary Table 3). In detail, the two basic residues of the HNH ββα motif, Lys862 and Lys866, formed alternative ionic interactions with the three acidic residues Glu370, Glu371 and Glu396 on REC1, respectively (Supplementary Figs. 9a and 10a). Meanwhile, Lys775, Arg778 and Glu779 (on HNH flanking linker 1, L1) competed for binding to Glu584, Asp585, Arg586 and Lys558 of REC3, respectively (Supplementary Figs. 9c and 10c). The HNH loop immediately preceding the ββα motif made numerous side chain and backbone hydrogen bonds with REC2, such as Asn831 with Thr249/Asn251, and Ser834 with Gly247/Thr249 (Supplementary Figs. 9b and 10b). Interestingly, Asp835 alone formed multiple hydrogen-bonds to one helical turn of Ser217, Lys218 and Ser219 on REC2. Additionally, Arg832 and/or Arg859 (on ββα motif) formed charged interactions with the REC2 Glu223 residue (Supplementary Figs. 9b and 10b). Lying on the long loop between the two β elements of the ββα motif, Gln844 and Lys848 were engaged to Glu60 on BH and Thr58 (on the loop linking BH and RuvC) via hydrogen-bond and ionic interactions, respectively (Supplementary Figs. 9d and 10d). Another adjacent residue Ser845 was implicated in hydrogen-bonding to the + 3 **P** of the tDNA, a position only 1-nt from the cleavable site (Supplementary Figs. 9e and 10e). Also, the HNH domain formed a number of polar contacts with the backbone of the sgRNA (primarily at its middle guide segment). Located on the N-terminal ββα motif flanking helices, the residue pair of Asn803 and Gln807, and the triplet of Arg780, Arg783 and Tyr812 firmly caught the two nucleotides **8** and **9** of the sgRNA (numbered **1** from the most PAM-distal end), respectively, through hydrogen-bonds and/or salt bridges (Supplementary Figs. 9 f and 10 f). Meanwhile, the two basic residues, Lys848 and Arg895 (on the last C-terminal ββα motif flanking helix) participated in ionic interactions with the trinucleotide stretch from sites **11** to **13** (Supplementary Figs. 9d and 10d). Along with Mg^2+^, the identified Cas9 residues above likely play a crucial role in locking the HNH domain onto the scissile phosphate on tDNA.

Importantly, the structural information derived here can be exploited to minimize the off-target effects of CRISPR-Cas9. Guided by the “excess energy” hypothesis that Cas9-sgRNA is more energetic than required for its optimal on-target recognition and cleavage, two recent publications^20,21^ reported several versions of high-fidelity Cas9 variants bearing multiple alanine substitutions, which were engineered based solely on an inactive DNA-bound crystal structure available at that time. We noticed that there are five basic residues on the HNH domain (viz. Lys775, Lys810, Arg832, Lys848 and Lys862) described here that have been experimentally tested (Supplementary Fig. 1c)^20^. Neutralization of these residues was demonstrated to improve Cas9 specificity in varying degrees. Specifically, the two single mutants Lys810Ala and Lys848Ala performed best, exhibiting remarkably reduced off-target cleavage at all tested sites while maintaining on-target efficiency^20^. From our catalytic Cas9 structure, Lys810 formed hydrogen-bonding/salt bridge interactions with +6 **P** of tDNA (Supplementary Fig. 9g), a location of two nucleotides from the scissile phosphate, while Lys848 was engaged to the residues on BH and sgRNA (Supplementary Figs. 9d and 10d). Based on these observations, the alanine substation of Lys810 or Lys848 could destabilize the activated conformation of HNH domain, thereby requiring more stringent canonical basing paring between the guide RNA and tDNA. With the new structural information, likewise, we believe that more Cas9 nucleases with enhanced specificity could be rationally designed by trying different single and combined mutations.

## Discussion

**Figure 5:**
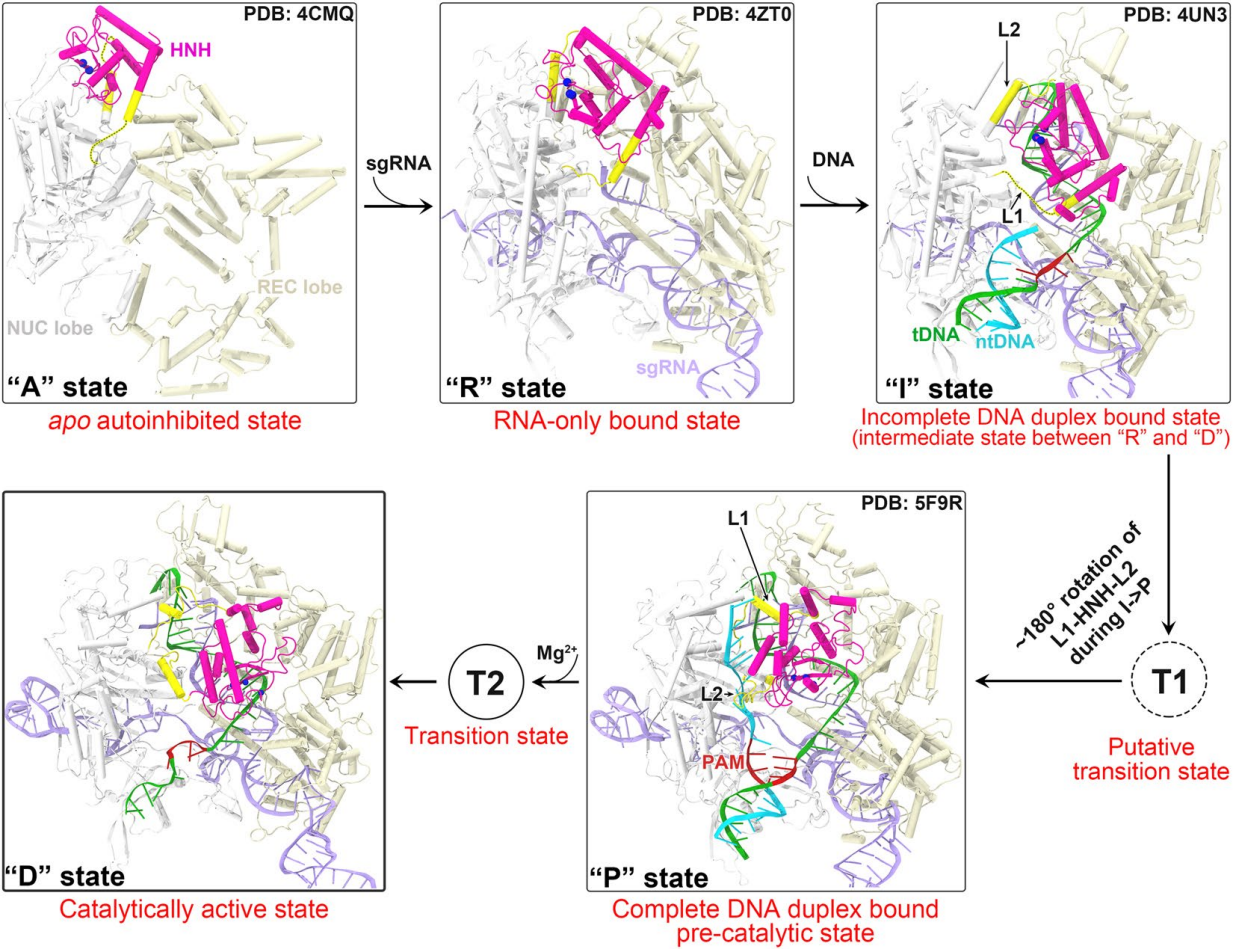
Conformational activation pathway of Cas9 HNH nuclease domain. The HNH domain and flanking liker regions (i.e. L1 and L2) are highlighted in magenta and yellow, respectively. The PAM is colored dark red and the three putative catalytic residues of HNH domain are represented as blue spheres. The dash lines denote the disordered liker loops. All of the solved Cas9 crystal structures in different binding forms assume an inactive state as for both RuvC and HNH nuclease domains. Using single-molecule FRET, Dagdas, Chen, *et al*.^22^ identified three distinct conformational states of Cas9, designated state “**R**”, “**I**” and “**D**”, respectively. In our study, with the involvement of Mg^2+^ and absence of ntDNA, we addressed how the HNH domain is “docked” toward the catalytically competent state. However, another fundamental question remains open to be answered that what factors trigger ~180° rigid-body rotation of HNH domain alongside its two flanking likers during **I**→**P** state transition. We propose that there likely exists a functionally important transition state (**T1**) between **I** and **P** states that acts a conformational checkpoint determining the fates (cleaved or not) of bound on- or off-target substrates.

Multiple Cas9 crystal structures in different binding forms have been solved over the past few years^7–10,14,16,17^; however, none of these assumes a functionally fully active state as for either of its two nuclease domains (Fig. 5). In our recent work, we reported the catalytically competent state of the Cas9 RuvC domain primed for cutting the ntDNA by molecular dynamics (MD) simulations^18^. Using two distinct sampling strategies, i.e., the biased tMD and unbiased cMD^ens^, we here obtained well-converged catalytic conformations for the HNH domain, especially in terms of HNH domain orientation (Fig. 1 and Fig. 3), Mg^2+^ coordination geometry (Fig. 2) and newly established interactions with HNH domain (Supplementary Figs. 9-10). The success of cMD^ens^ here can be ascribed to: i) enhanced flexibility of the HNH domain by removal of ntDNA (Fig. 1 and Supplementary Figs. 2-3); ii) Mg^2+^-mediated electrostatic attraction at the binding interface (Figs. 2 and 4); and iii) favorable charged and polar interactions between the HNH domain and other components (Supplementary Figs. 9-10). Apparently, these factors substantially lower the energetic barrier between the pre-catalytic and catalytic states, thereby making it possible for a large conformational change of the HNH domain to occur (Fig. 3 and Supplementary Fig. 8 and Supplementary Table 2) could be accessible within dozens of microseconds (Table 1). The cMD^ens^-based sampling approach might be applied to other systems provided that the relevant conformational transition pathway can be defined. The mechanism of conformational control over the spCas9 HNH domain is likely to be broadly conserved across diverse Cas9 family members, given their similarities in structure and function^7–10,14,16,17^.

In order to enhance the conformational sampling of the HNH domain and obtain the active state conformation in a relatively shorter time-scale that is reachable by MD simulations, the ntDNA was not included in our simulations. Several experiments^5,15,35^ have unambiguously shown the cleavage of a single tDNA substrate in the absence of ntDNA, suggesting that the Cas9 HNH domain is able to reach the active state with tDNA alone, as we demonstrated here. Even though we removed the ntDNA to facilitate our simulations, we do not exclude the potentially important roles that the ntDNA may play in the catalytic conformation activation of Cas9. One possible role of the ntDNA is to stabilize the activated conformation by making interactions with the surface residues on the HNH domain^7,15^. For instance, a recent computational study identified a stable hydrogen-bond formed between the ntDNA and Lys913, a residue on the linker 2 (L2) immediately succeeding the C-terminus of the HNH domain^28^. Another important role of the ntDNA could be its effects on the cleavage kinetics: without the entire ntDNA or the PAM sequence on it, the single tDNA substrate was cleaved about two orders of magnitude slower than the dsDNA substrate, despite comparable binding affinities of both substrate types to Cas9-gRNA^35^. Given the observation that Cas9-gRNA rapidly dissociates from non-PAM DNA, we reason that the ntDNA accelerates the reaction rates probably by promoting the HNH domain rotation during strand unwinding (Fig. 5) and/or by facilitating rapid interrogation and loading of the DNA target via PAM recognition, which could be a rate-limiting step that influences overall cleavage reaction^35^. Meanwhile, another possibility may exist wherein the ntDNA PAM acts as an allosteric regulator of Cas9 nuclease activity that contributes to concerted cleavage of both DNA strands^15,35,36^. In the presence of ntDNA, the Cas9 catalytic state might adopt a somewhat different conformation from that captured here. Yet, the global orientation of HNH domain relative to the REC lobe and tDNA, and the coordination configuration at the binding interface should vary little. In this work, we have provided the missing link of how the HNH domain transitions from the pre-catalytic to catalytic state. However, another fundamental question remains open to be answered. Specifically, what factors trigger an approximately 180° rigid-body rotation of L1-HNH-L2 during the previously identified immediate (“**I**”) to pre-catalytic (“**P**”) state transition (Fig. 5)? We propose that there likely exists a functionally relevant transition state between the **I** and **P** states, which acts a conformational checkpoint determining the fates (cleaved or not) of bound on- or off-target substrates. By introducing a certain number of mismatches, this state might be captured through smFRET or crystallography or identified with molecular dynamics free energy simulations^19^.

The two distinct conformational activation pathways for the HNH domain, implemented respectively by tMD and cMD^ens^, strongly suggest Mg^2+^ is indispensable for the formation and stability of the catalytic state. In the absence of Mg^2+^, it is conceivable that the HNH domain swings repeatedly toward and away from the tDNA but fails to engage an active conformation (Fig. 4), as demonstrated by the smFRET experiments^22,23^. If Mg^2+^ diffuses into the binding interface, the HNH domain readily docks onto and gains the stable association with the opposite tDNA, accompanying new interactions formed with other components (especially REC lobe and sgRNA) in the system. Therefore, beyond its catalytic role, Mg^2+^ also acts as a facilitator and stabilizer of the functional conformational state. Combining these results with our previous study with the Cas9 RuvC domain^18^, we hold that the roles of Mg^2+^ revealed here are common in other divalent metal ion dependent nucleases^11,13^. Besides Mg^2+^, other metal ions like Mn^2+^, Ca^2+^ and Co^2+^ are also able to activate the HNH conformation and stabilize its catalytic state^5,22^. These ions can assume a similar octahedral coordination geometry and a comparable effective radius to that of Mg^2+^ as observed here (Fig. 2)^37^. Intriguingly, Co^2+^ does not support HNH nuclease activity^5,22^. Hence, the catalytic conformation might be crystalized with wild-type Cas9 and Co^2+^. This strategy could be more effective than using Cas9 nickase mutants and Mg^2+^, as the active residue substitution inevitably destabilizes the enzyme/substrate complex.

The derived catalytic state provides a different perspective on the sources of enhanced Cas9 specificity through alanine mutagenesis. The five basic residues of the L1 linker and HNH domain, Lys775, Lys810, Arg832, Lys848 and Lys862, whose single alanine substitution was shown to reduce Cas9 off-target effects (Supplementary Fig. 1c), were previously supposed to make contacts with the phosphate backbone of the ntDNA^20^. Based on our simulations, Lys775, Arg832 and Lys862 form ionic/hydrogen-bonding interactions with the negatively charged residues on the REC3 (Glu584 and Asp585), REC2 (Glu223) and REC1 (Glu370 and Glu396) domain, respectively, while another residue Lys848 is simultaneously engaged to the residues on BH (Thr68 and Glu60) and the sgRNA backbone (Supplementary Figs. 9-10 and Supplementary Table 3). Apparently, these new interactions directly contribute to HNH domain docking onto tDNA, and neutralization of the basic residues could destabilize formation of the active HNH conformation, thereby requiring more stringent Watson-Crick base pair complementarity with the sgRNA. This view is in contrast with the hypothesis that the improved specificity exclusively results from diminished interactions with the ntDNA^20^. Remarkably, our catalytic model affords additional molecular cues as to why the identified Cas9 variants^20^, K810A/K1003A/R1060 and K848A/K1003A/R1060 [referred to as eSpCas9(1.0) and eSpCas9(1.1), respectively], exhibit genome-wide high editing specificity. For eSpCas9(1.0), its specificity improvement is due to attenuated interactions with both DNA strands, whereas for eSpCas9(1.1), its improved specificity is rooted in a combined effect involving simultaneous weakened binding with the two DNA strands, sgRNA and Cas9 BH. Meanwhile, we highlight that it cannot be ruled out that the basic residues of the HNH domain change interacting partners (e.g., from ntDNA to tDNA) during different stages of conformational activation, given the striking flexibility of HNH domain (see Fig. 5). Moreover, our model can also explain the decrease in specificity upon converse Ser845Lys replacement^20^, which arises from the strengthened interaction of the HNH domain with the tDNA backbone at a position only 1-bp from the cleavage site (Supplementary Figs. 9e and 10e).

In the framework of the “excess energy” hypothesis proposed for Cas9-sgRNA^20,21^, likewise, the new structural information here can be exploited to rationally design additional Cas9 variants with improved specificity. After careful inspection of the locations of the identified residues and their interactions within the whole complex, we suggest more than a dozen of potential mutation sites for future tests (See Supplementary Table 3). Through further integration with previously screened candidate sites, we believe that different versions of high-fidelity Cas9 mutants could be customized specifically for minimizing the off-target effects occurring at the PAM proximal or distal ends, or even at the non-standard repetitive sites. As there is no one versatile Cas9 nuclease capable of eliminating all sorts of off-target cleavage, such an approach would be of worthy of investigation. We are collaborating with the experimental groups to test our predictions, and the results would be reported in due time.

In summary, herein we have reported a cross-validated catalytically active model of the Cas9 HNH nuclease domain poised for cutting the tDNA and demonstrated the essential roles of divalent metal ions in facilitating and stabilizing the formation of the active conformation. More importantly, the derived catalytic state provides novel structural information for Cas9 specificity enhancement. Further studies on more different conformational states as well as the binding and cleavage mechanism of Cas9 would contribute to the additional refinement of the CRISPR-Cas9 genome-editing toolbox.

## Materials and Methods

### System Setup

The initial configurations of the two Cas9 complex systems, viz. Cas9-sgRNA-dsDNA (with tDNA) and Cas9-sgRNA-tDNA (without ntDNA) were derived from the recently solved crystal structure at 3.4 Å resolution [PDB accession code: 5F9R^7^]. We note that the ntDNA herein refers to the whole non-target DNA strand encompassing the 5′-end protospacer sequence, protospacer-adjacent-motif (PAM), and 3′-end flanking sequence. The ntDNA-free system was built by removing the entire non-target DNA strand from the intact structure, while for the dsDNA-bound system, the ntDNA 5′-end cleavage product was excluded based on our previous study^18^. Following the two-metal-ion and one-metal-ion mechanisms proposed for Cas9^7,9,14^, two Mg^2+^ were placed around the RuvC active center with partial ntDNA or without ntDNA, and if applicable, one Mg^2+^ was introduced at the HNH active center (Table 1), as previously described^18^. The missing heavy atoms and hydrogen atoms were added using *leap* program within AmberTool16^38^ and the protonation states of protein titratable residues were assigned through the on-line tool H ++ at a physiological pH of 7.5^39^, followed by visual check. Each system above was then immersed in a cubic water box with a thickness of 13.5 Å, leading to a simulation cell of approximately 139 × 124 × 187 Å^**3**^. To mimic the reaction buffer^5,14,15,35^, extra 7 or 8 Mg^2+^ were added into the water box to yield a concentration of 5 mM, and the ionic strength of KCl was set to 100 mM. The total atoms of Cas9-sgRNA-dsDNA and Cas9-sgRNA-tDNA solution systems add up to ~283,500 and ~281,800, respectively.

### Conventional Molecular Dynamics Simulations

All kinds of simulations were performed by the GPU version of AMBER16 *pmemd* engine (*pmemed*.*cuda*)^38^ except the targeted MD simulations that were realized with NAMD2.10^40^ (as described below). The amber force fields ff14SBonlysc, ff99bsc0 and ff99bsc0_chiOL3 were used to describe paired interactions involving protein, DNA and RNA, respectively. The TIP3P model^41^ was selected for water and the recently developed ion parameter sets optimized in TIP3P water were employed for the mono- and divalent ions^42,43^. It should be mentioned that none of the available non-bonded models for metal ions, especially the multivalent ions, is able to reproduce various experimental properties simultaneously^44^; the Mg^2+^ parameter set here, as we previously used for the same enzyme^18^, represent the best possible compromise targeting the experimental coordination number, Mg^2+^-O distance and hydration free energy^43^. The short-range non-boned interactions were truncated at 10 Å, and the long-range electrostatics were treated via the particle mesh Eward summation (PME) method^45^ using a grid spacing of 1 Å. The bonds involving hydrogens were constrained through the SHAKE algorithm^46^. Each system was subjected to a thorough energy minimization with the solute heavy atoms constrained, then followed by slow heating from 0 K to the target 310.15 K and 10-ns equilibration in the isothermal-isochoric (NVT) ensemble in which the backbone atoms were restrained. Finally, the production simulations (i.e. **G1**, **G2** and **G10** in Table 1) without any restraints were conducted under the isothermal-isobaric (NpT) condition and each independent run was extended to at least 1000 ns. The temperature was maintained at 310.15 K through the Langevin thermostat and the pressure was controlled at 1.013 bar via the Monte Carlo barostat. The integration time step was set to 1 fs during minimization and equilibration, and 2 fs in the production stage. The trajectory snapshots were saved at 10-ps intervals for analysis.

### Accelerated Molecular Dynamics

aMD is an enhanced sampling technique by adding a non-negative potential [$\triangle V({\boldsymbol{r}})$] to the original potential energy surface [$V({\boldsymbol{r}})$] when it falls below a threshold energy ($E$), as

1 $${\rm{\Delta }}V({\boldsymbol{r}})=\{\begin{array}{cc}0 & V({\boldsymbol{r}})\ge E\\ \frac{{(E-V({\boldsymbol{r}}))}^{2}}{\alpha +(E-V({\boldsymbol{r}}))} & V({\boldsymbol{r}}) < E\end{array}$$

where the acceleration factor $\alpha$ modulates the depth and local roughness of the energy basins in the modified potential^29,30^. Apparently, this simple formalism has several practical advantages: only two parameters ($E$, $\alpha$) need to be specified and an *a prior* reaction coordinate is not required to be defined. Here, two acceleration levels were applied to the Cas9-sgRNA-ntDNA system, i.e. boosting only the dihedral energy terms (dihedral aMD) and boosting the whole potential with an extra boost to the dihedrals (dual aMD) (**G3** and **G4**, Table 1). Following previous works^30,47^, the boosting parameters for each aMD run were estimated from the corresponding 60-ns conventional MD simulations carried out in the NVT ensemble. The aMD simulations were started from the last snapshots of the above short cMD simulations and were performed also in NVT ensemble, lasting 650 ns and 1000 ns for the dihedral and dual modes, respectively (**G3** and **G4**, Table 1). In our preliminary tests, we also ran the new variant GaMD (Gaussian accelerated MD)^48^ that allows for improved reweighting. In results, we found appreciable loss of protein secondary structures, thereby not applying this approach herein.

### Targeted Molecular Dynamics

tMD induces conformational transition between two known states by means of steering forces^33,34^. At each time step, the root-mean-square deviation (RMSD) between the current coordinates and the target structure is calculated. The force exerted on each atom is given by the gradient of the potential,

2 $${U}_{tMD}=\frac{1}{2}\frac{k}{N}{[RMSD(t)-RMS{D}^{\ast }(t)]}^{2}$$

where the spring constant $k$ is scaled down by the number $N$ of targeted atoms, ${RMSD}(t)$ is the instantaneous best-fit RMSD of the current coordinates from the target conformation, and ${{RMSD}}^{\ast }(t)$ evolves linearly from the initial RMSD at the first tMD step to the final value at the last step. The two start structures for tMD were extracted from the replicated long cMD simulations (Table 1), based on the HNH domain closeness to the putative catalytic state modeled from the crystal structure of T4 endonuclease VII (Endo VII) complexed with a DNA Holliday junction (see Supplementary text and Supplementary Fig. 5)^26^. The guiding forces were imposed only on the backbone atoms of HNH domain. The initial RMSDs of the biased atoms from the target states are around 10 Å, which are significantly lowered compared with that of 25 Å calculated directly from the pre-catalytic state structure. With the TclForces functionality in NAMD, we used an in-house TCL (Tool Command Language) script to implement the mass-weighted partial tMD simulations. The source code is available upon request. During tMD, the Cα atoms of the protein residues (excluding HNH domain) exhibiting low fluctuations was weakly restrained with a force constant of 0.1 kcal/mol/Å^2^ to prevent solute drift. Based on our previous experience^49^, a small force constant of 0.25 kcal/mol/Å^2^ per targeted atoms was adopted, and the simulation length reached up to 100 ns, representing a decreasing rate in RMSD of ca. 0.1 Å per ns. The tMD simulations were performed in NVT ensemble with a time step of 1 fs. The above procedure could ensure a least perturbation on the system resulting from external forces applied by tMD (see more details in Supplementary Information).

### Post Targeted Molecular Dynamics Simulations

At the end of tMD, the RMSD difference reduced to ~0.8 Å, indicating completion of the expected conformational transition. Two trajectory snapshots at ~90 ns of the above parallel tMD (**G5**, Table 1) were then extracted and subjected to 50-ns equilibration with gradually released restraints on the protein backbone atoms. The final structures were used to seed subsequent unbiased MD simulations (**G6**, Table 1), in which one Mg^2+^ was introduced at between the HNH active site and the ntDNA scissile phosphate according to the one-metal-ion mechanism. Each run was extended to 800 ns (**G6**, Table 1). Here, we did not employ the tMD end structures (i.e. at 100 ns) as the start points for Mg^2+^ introduction, given that the modeled target coordinates used in tMD do not necessarily represent a true catalytic state, and importantly, that the Mg^2+^ might assist further conformation change to bridge the distance gap for catalysis as we previously demonstrated^18^. This consideration allowed for spontaneous adaptation of the system to the catalytic conformation, thereby eliminating the potential artifacts from tMD. To probe the role of Mg^2+^, we proceeded to perform a set of conventional simulations started from the derived catalytic state, in which the above placed Mg^2+^ was moved from the active center to the bulk solution (**G7**, Table 1).

### Trajectory Analysis Methods

Details of principal component analysis (PCA), cluster analysis, binding free energy and non-bonded interaction energy calculations and other analyses were presented in Supplementary Information.

## Electronic supplementary material

Supplemental information
