# Supplementary material for: Structure and Dynamics of Cas9 HNH Domain Catalytic State
Source: Sci Rep. 2017 Dec 8;7:17271. doi: 10.1038/s41598-017-17578-6 (PMC5722908; doi:10.1038/s41598-017-17578-6)
Supplement: Supplementary file 1 — Supplemental information [file 41598_2017_17578_MOESM1_ESM.pdf]

Supplementary Information for

Structure and Dynamics of Cas9 HNH Domain Catalytic State

*Zhicheng Zuo and Jin Liu**

Department of Pharmaceutical Sciences, University of North Texas Systems College of Pharmacy, University of North Texas Health Science Center, Fort Worth, TX 76107

*Correspondence author: Jin Liu, jin.liu@unthsc.edu

Table of Contents

I. Supplementary Figures [10]

II. Supplementary Tables [3]

III. Supplementary Text

a. Principal Component Analysis

b. HNH Active State Modeling & HNH Pairwise RMSD Computation

c. Details of Generating tMD-derived Catalytic State

d. Details of Generating cMD^{ens}-derived Catalytic State

e. Cluster Analysis

f. Binding Free Energy & Per-residue Energy Decomposition

g. Non-bonded Interaction Energy Calculation

VI. Supplementary References

I. Supplementary Figures

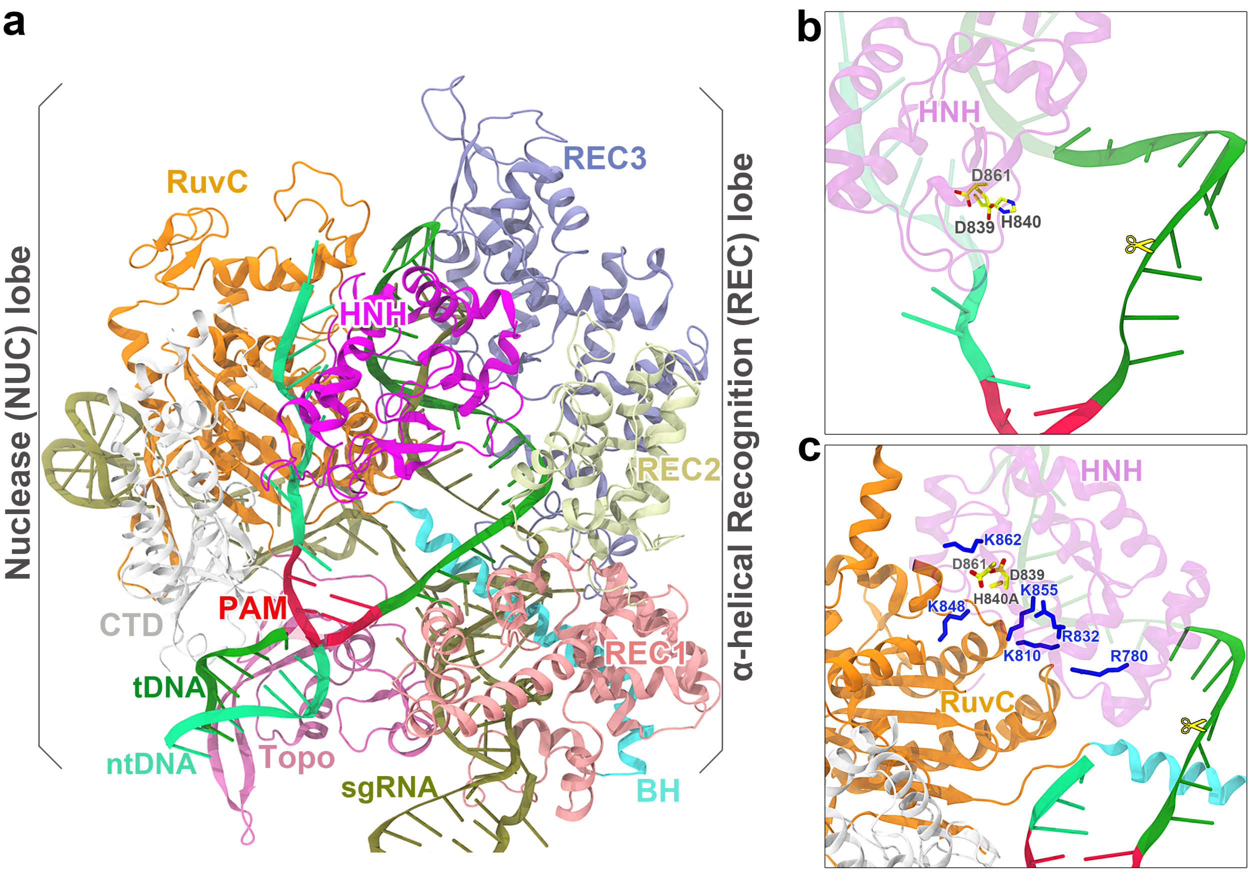

Figure S1. spCas9-sgRNA complexed with PAM-containing double-stranded DNA (dsDNA) substrate. (**a**) Overall architecture of the ternary complex of spCas9, sgRNA and dsDNA (PDB code: 5F9R). Cas9 NUC lobe comprises of two nuclease domains (RuvC and HNH), C-terminal domain (CTD) and topoisomerase homology (Topo) domain, and REC lobe is spatially divided into three domains (REC1, REC2 and REC3); the two lobes are connected by an arginine-rich (bridge) helix (BH). The target and non-target DNA strands (tDNA and ntDNA) are colored dark and light green, respectively, with the PAM duplex highlighted in crimson. (**b**) Close-up view of the HNH domain active center (PDB code: 5F9R). The putative catalytic residues are depicted in a stick model and colored by atom type (C, yellow; N, blue; O, red). (**c**) Structured-guided protein engineering to improve spCas9 specificity (PDB code: 4UN3). Neutralization of the selected basic residues on the HNH domain (colored blue) was shown to reduce spCas9 off-target effects while maintaining off-target activity. The cleavage site on tDNA is denoted with a scissor.

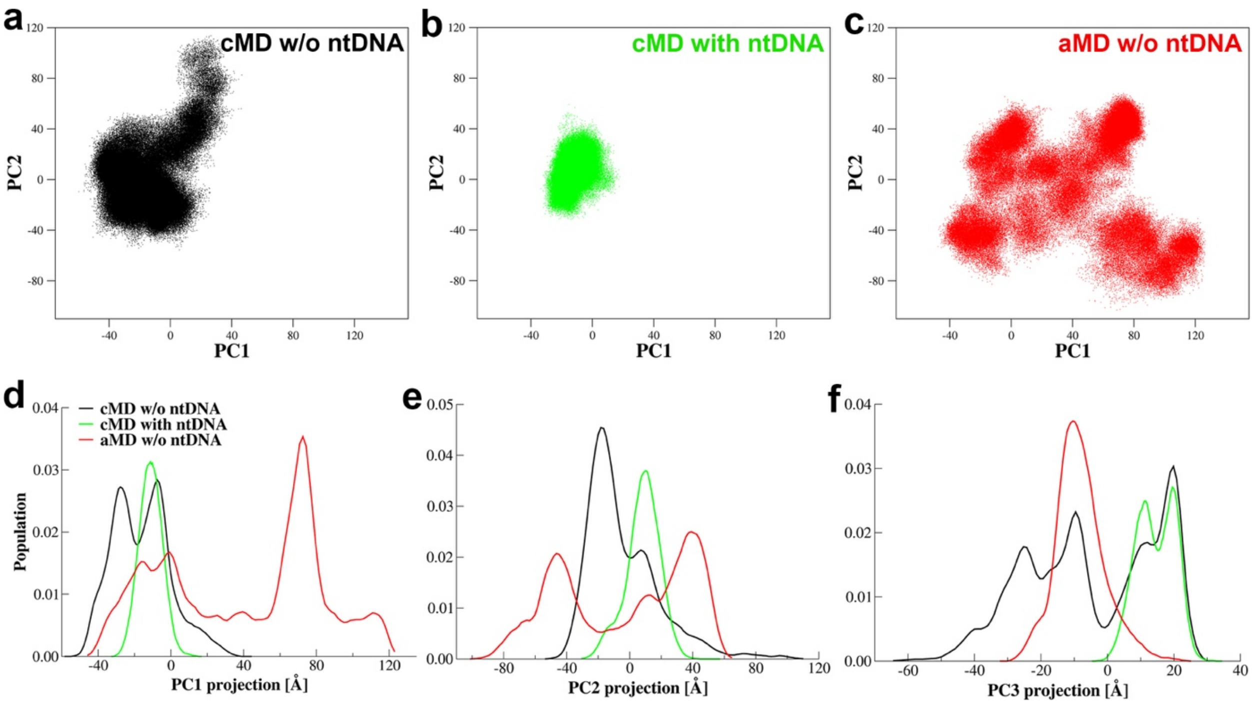

Figure S2. (a-c) Projections of the conventional MD simulations without ntDNA (cMD w/o ntDNA) (**a**), conventional MD simulations with ntDNA (cMD with ntDNA) (**b**) and accelerated MD simulations without ntDNA (aMD w/o ntDNA) (**c**) onto the first two eigenvectors calculated from the whole trajectories for the HNH domain. (**d-f**) overlap of the histograms of the first (**d**), second (**e**) and third (**f**) PC projections for the conventional MD simulations without ntDNA (cMD w/o ntDNA, black line), conventional MD simulations with ntDNA (cMD with ntDNA, green line) and accelerated MD simulations without ntDNA (aMD w/o ntDNA, red line) and. See also **Figure 1**.

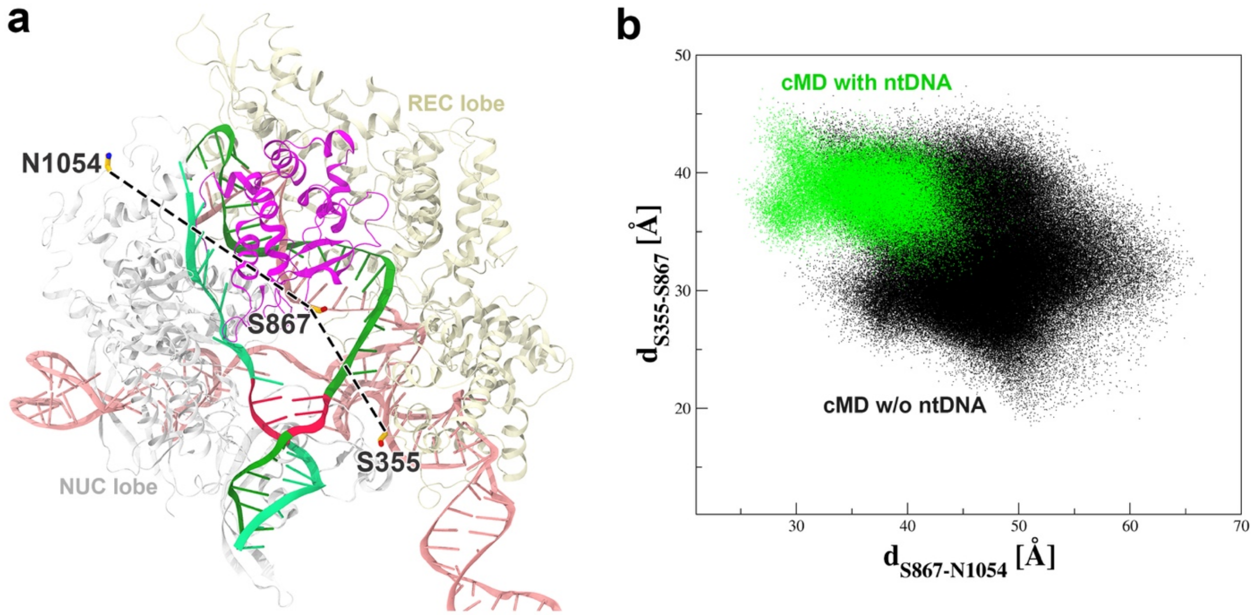

Figure S3. FRET labeled residue pairs shown with 5F9R (a) and scatter plot of the distances for the labeled residue pairs calculated from conventional MD simulations without ntDNA (cMD w/o ntDNA, black dots) and with ntDNA (cMD with ntDNA, green dots). Ser355, Ser867 and Asn1054 are located in the REC1, HNH and RuvC domains, respectively. These residues were previously selected to characterize different conformational states of HNH domain in FRET experiments[1-3].

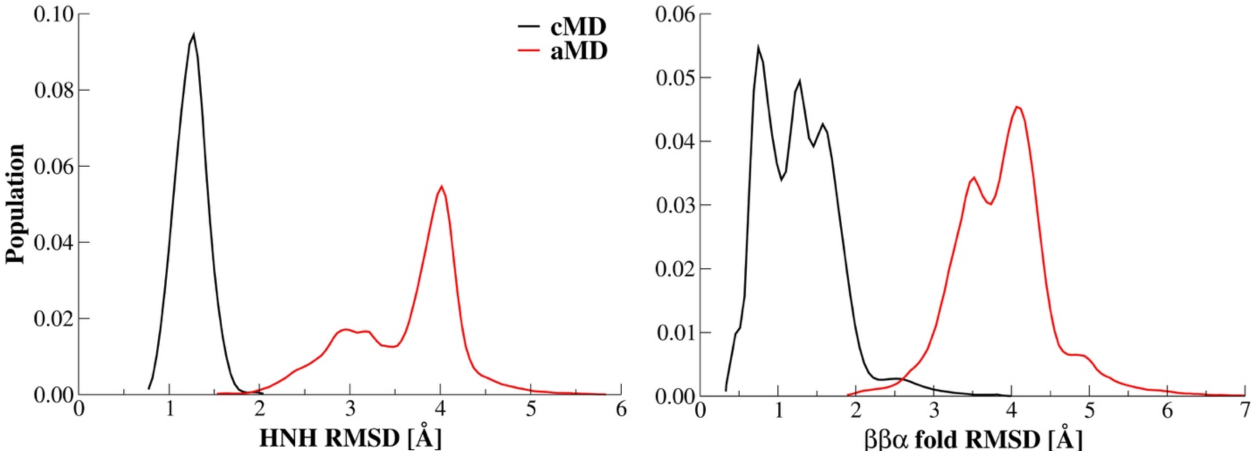

Figure S4. Cα RMSD distributions for the HNH and ββα fold calculated from the conventional and accelerated MD simulations relative to the starting crystal structure (PDB code: 5F9R). The average pairwise RMSDs for the HNH domain and ββα motif among the available Cas9 crystal structures in different binding forms is 1.4±0.6 and 1.4±0.7 Å, respectively (see **Table S1**), which are comparable to the corresponding peak values calculated from the cMD simulations. In contrast, aMD shows significantly larger RMSD values peaking at 4 Å, indicating the enhanced sampling accompanies considerable internal structural change.

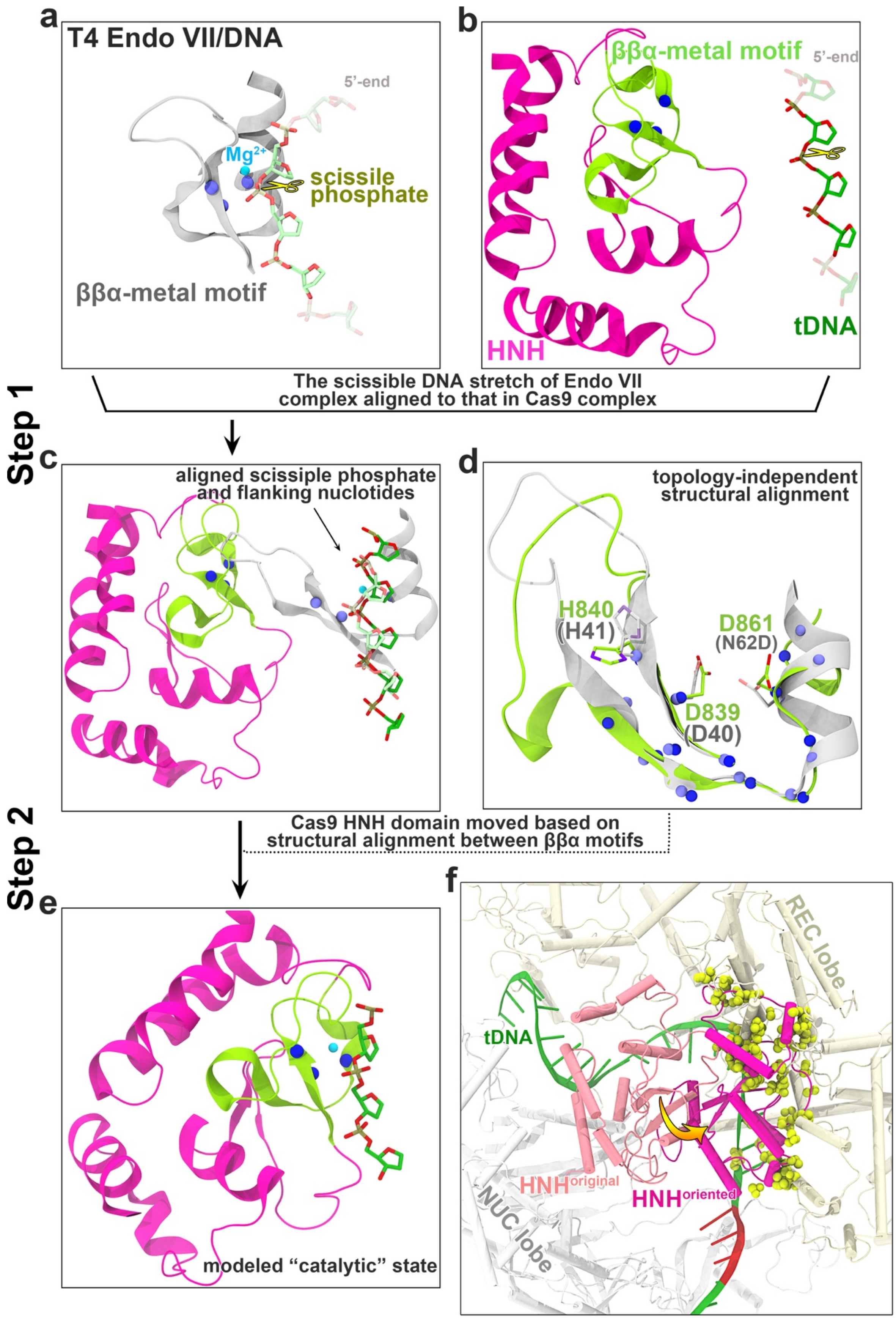
a
T4 Endo VII/DNA
5'-end
Mg2+
scissile phosphate
ββα-metal motif
b
ββα-metal motif
5'-end
HNH
tDNA
The scissible DNA stretch of Endo VII complex aligned to that in Cas9 complex
Step 1
c
aligned scissiple phosphate and flanking nuclotides
d
topology-independent structural alignment
H840
(H41)
D861
(N62D)
D839
(D40)
Cas9 HNH domain moved based on structural alignment between ββα motifs
Step 2
e
modeled "catalytic" state
f
REC lobe
tDNA
HNHoriginal
HNHoriented
NUC lobe

Figure S5. Putative catalytic state of Cas9 HNH domain modeled from T4 Endonuclease VII (Endo VII)/DNA complex (PDB code: 2QNC). (**a**) T4 Endo VII ββα-metal motif complexed with a DNA substrate. The Cα atoms of the active residues (i.e. Asp40, His41 and Asn62) are rendered as light blue spheres, and the coordinated Mg^{2+} is depicted as a cyan sphere. (**b**) Cas9 HNH domain opposite to the target DNA strand (PDB code: 5F9R). The Cα atoms of the putative catalytic residues (i.e. Asp839, His840 and Asp861) are represented as dark blue spheres and the HNH domain ββα-metal motif is colored yellow-green. (**c**) The scissile phosphate and flanking nucleotides of the DNA substrate in **a** superimposed onto the corresponding stretch in **b** alongside the ββα-metal motif. (**d**) Topology-independent structural alignment between Cas9 and Endo VII ββα-metal motifs (PDB codes: 5F9R and 2QNC) using the CLICK algorithm[4] The Cα RMSD of the equivalent residues (shown as spheres) between the two nucleases is 1.2 Å. The catalytic residues appear to be spatially superimposed well. (**e**) Cas9 HNH domain oriented toward the target DNA strand based on the transformation matrix obtained from **d**. (**f**) Direct "docking" of the HNH domain starting from the pre-catalytic state (PDB code: 5F9R) results in a number of steric clashes with other components (including Cas9 REC lobe, tDNA and sgRNA) in the system. The overlapping heavy atoms are shown as van der Walls spheres, using a distance cutoff of 1.4 Å. The backbone RMSD for the HNH domain between the pre-catalytic and resulting docked conformations is 25 Å here.

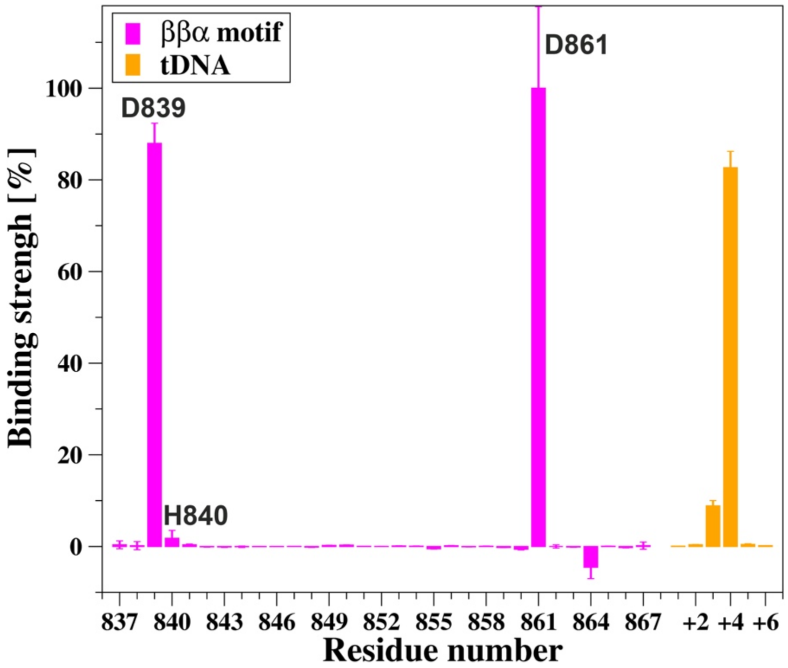

Figure S6. Relative binding strength of the residues on the HNH ββα fold and opposite tDNA with the coordinated Mg^{2+} computed via MM-GBSA approach. The energetic contribution of each residue is relative to Asp861 being of 100% binding strength. Positive and negative values indicate favorable and unfavorable binding, respectively.

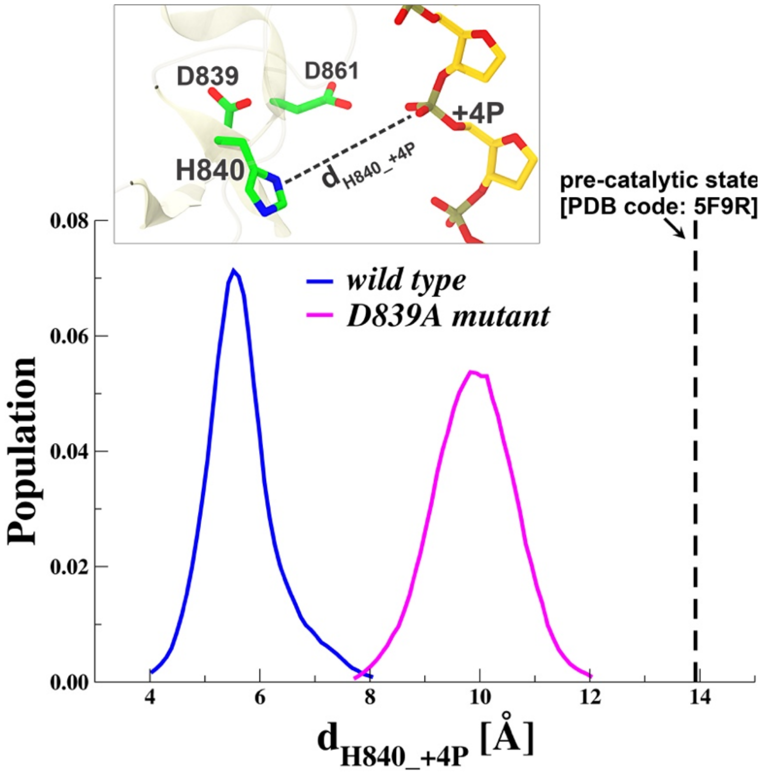

Figure S7. Distribution of the minimum distance ($d_{H840\_+4\mathbf{P}}$) between the general base His840 and scissile phosphate (+4**P**) calculated from the wild type and Asp839Ala mutant simulations. The last 300-ns of the simulation trajectories were collected for computations. The above inset illustrates the calculated distance and the black dashed line denotes the corresponding value from the pre-catalytic crystal structure. It is evident that upon Asp839Ala substitution, the general base His840 goes away from the scissile phosphate (from ~5 Å to ~10 Å), leading to impaired nuclease activity.

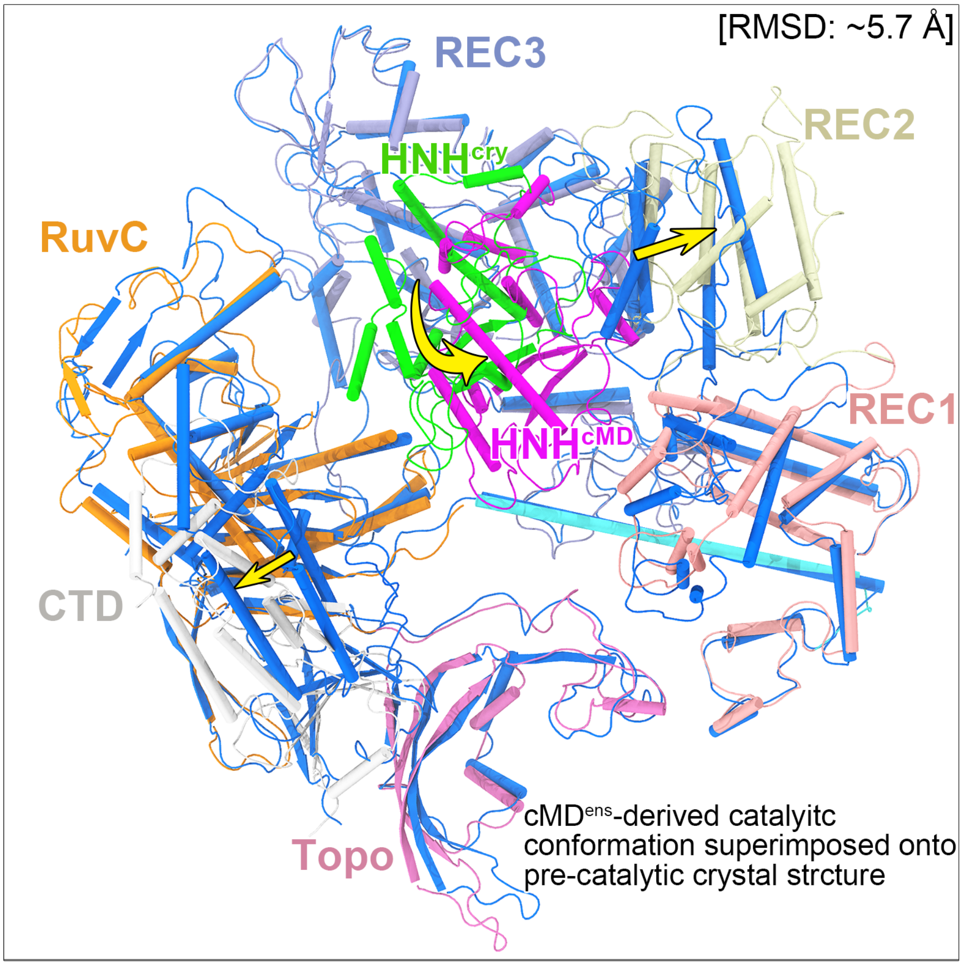

Figure S8. Structural superposition between the cMD^{ens}-derived catalytic state and crystal pre-catalytic state (PDB code: 5F9R). The HNH domain within 5F9R (labeled as HNH$^{\mathbf{cry}}$) is highlighted in green and the remainder of Cas9 colored blue, with the largest domain movement (involving HNH, REC2 and CTD) denoted by a yellow arrow. The cMD^{ens}-derived catalytic structure is colored by domains as in **Figure 3c**: HNH (labeled as HNH^{cMD}), magenta; REC1, pink; REC2, pale yellow; REC3, iceblue; RuvC; orange; CTD; grey; Topo, hotpink, BH, cyan.

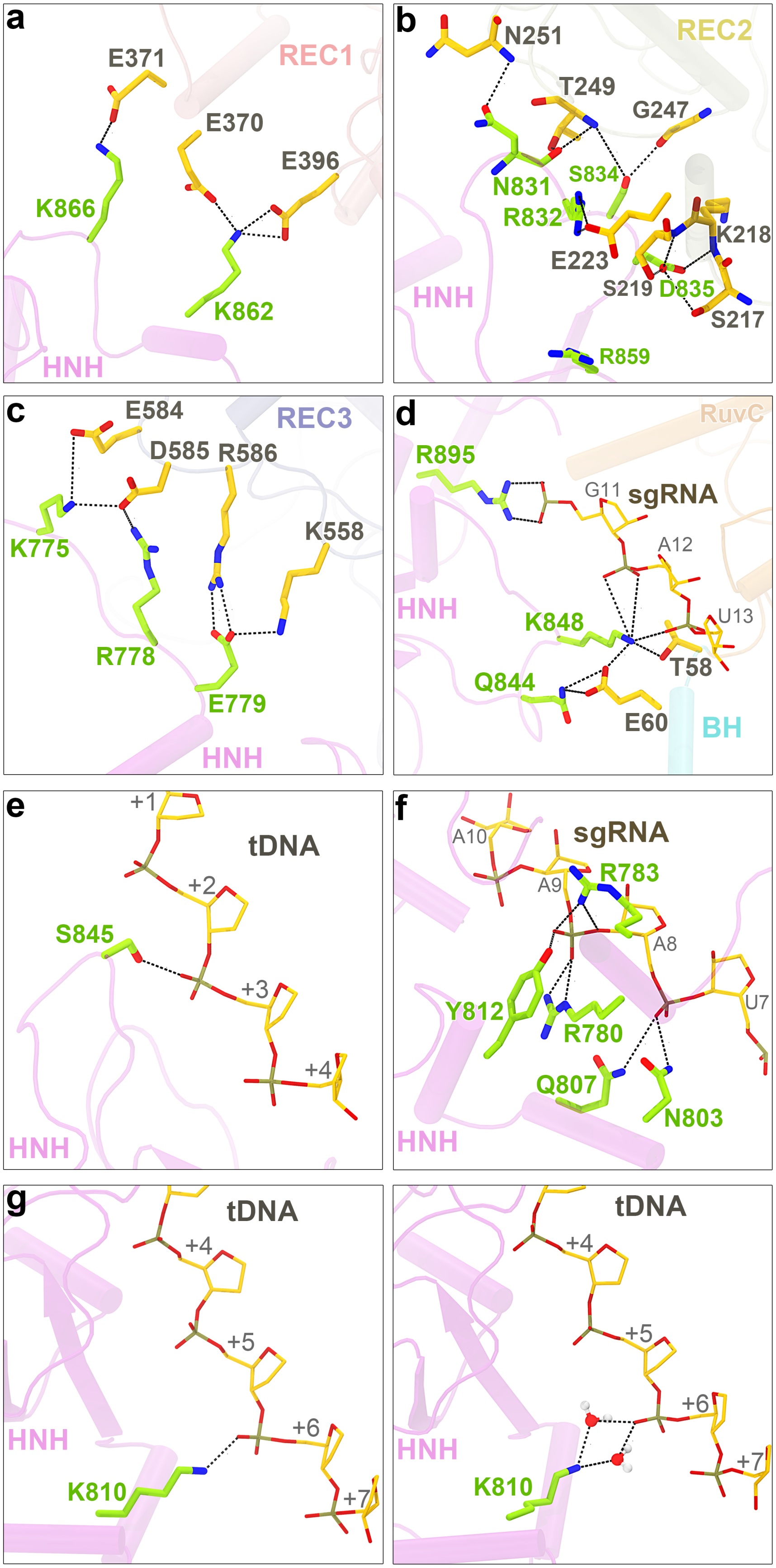

a
E371
REC1
E370
E396
K866
K862
HNH
b
N251
REC2
T249
G247
N831
S834
R832
K218
E223
S219
D835
S217
HNH
R859
c
E584
REC3
D585
R586
K558
K775
R778
E779
HNH
d
RuvC
R895
G11
sgRNA
A12
U13
HNH
K848
T58
Q844
E60
BH
e
+1
tDNA
+2
S845
+3
+4
HNH
f
A10
sgRNA
R783
A9
A8
U7
Y812
R780
Q807
N803
HNH
g
tDNA
+4
+5
+6
+7
HNH
K810
tDNA
+4
+5
+6
+7
HNH
K810

Figure S9. New interactions established between the catalytic HNH domain and other components in the complex system identified from the post target-MD (tMD) simulations. (**a**) Interactions with the REC1 domain. (**b**) Interactions with the REC2 domain. (**c**) Interactions with the REC3 domain. (**d**) Interactions with the bridge helix (BH) and sgRNA. (**e,g**) Interactions with the tDNA. (**f**) Interactions with the sgRNA. The HNH domain residues are highlighted in green. The dash lines denote the salt bridges and/or hydrogen bonds. Due to space limit, only interacting pairs with relatively high occupancy throughout the simulations are shown here, and the complete residue list is present in **Supplementary Table S3**. This Figure is comparable to **Supplementary Fig. 10**.

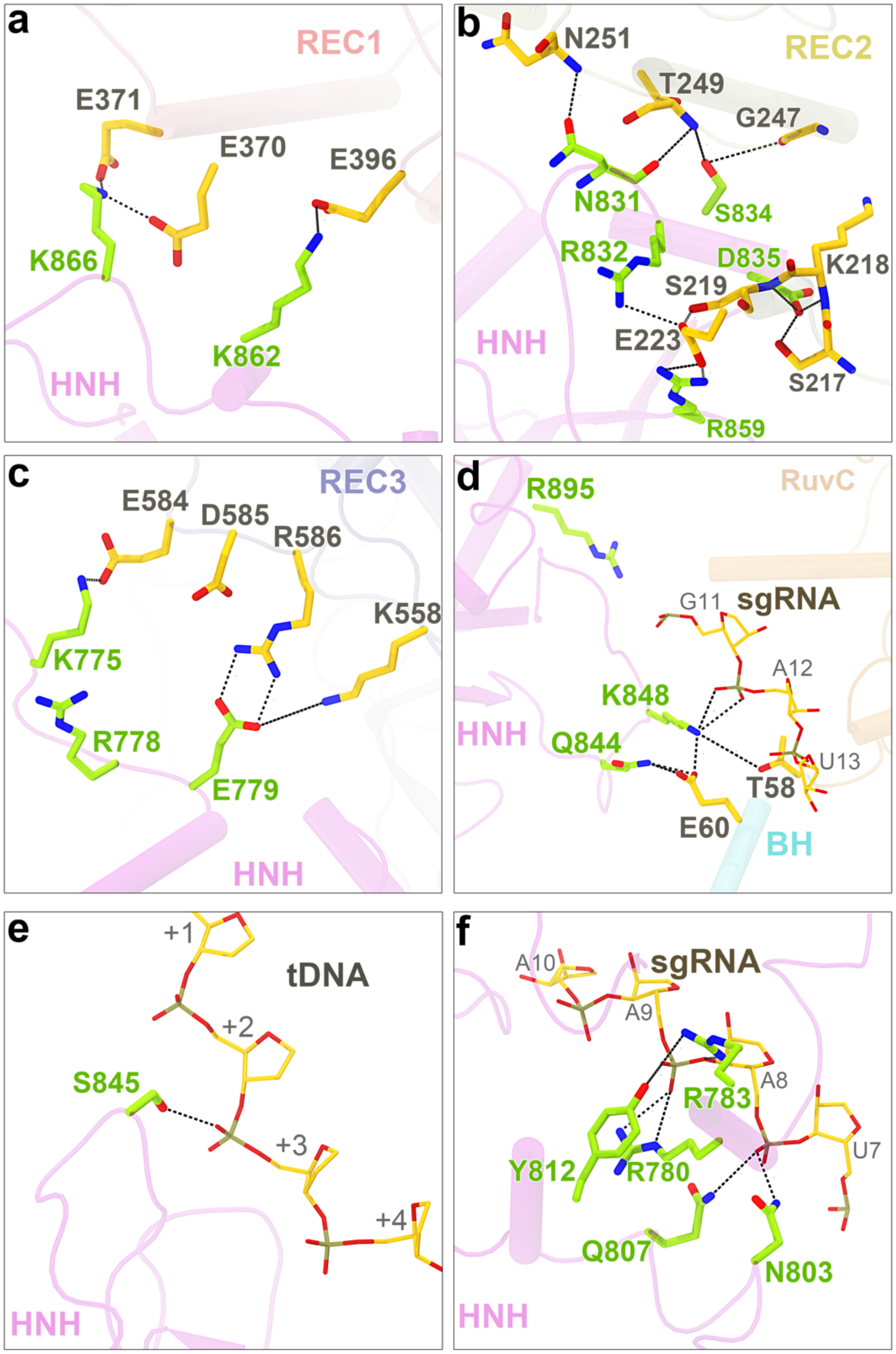

Figure S10. New interactions established between the catalytic HNH domain and other components in the complex system identified from the conventional ensemble MD (cMD^{ens}) simulations. (**a**) Interactions with the REC1 domain. (**b**) Interactions with the REC2 domain. (**c**) Interactions with the REC3 domain. (**d**) Interactions with the bridge helix (BH) and sgRNA. (**e**) Interactions with the tDNA. (**f**) Interactions with the sgRNA. The HNH domain residues are highlighted in green. The dash lines denote the salt bridges and/or hydrogen bonds. The interactions involving Lys810 are the same to that shown in **Supplementary Fig. 9g**. Note that all the interacting pairs do not necessarily appear in one single snapshot, and the complete residue list is present in **Table S3**.

II. Supplementary Tables

Table S1a | Pairwise RMSDs for the Cα atoms of HNH domain† among different Cas9 crystal structures [mean = 1.4 (0.6) Å]

PDB code	apo-Cas9			Cas9-sgRNA			Cas9-sgRNA-DNA		
	4CMP_A	4CMP_B	4CMQ_A	4ZT0_A	4ZT0_C	4ZT9_A	4OO8_A	4UN3_B	5F9R_B
4CMP_A*		0.5	0.5	1.9	2.4	1.8	1.8	1.8	1.8
4CMP_B	0.5		0.5	1.9	2.5	1.9	1.9	1.9	1.9
4CMQ_A	0.5	0.5		1.7	2.3	1.7	1.7	1.7	1.7
4ZT0_A	1.9	1.9	1.7		1.5	0.6	0.7	0.7	0.8
4ZT0_C	2.4	2.5	2.3	1.5		1.6	1.6	1.5	1.6
4ZT9_A	1.8	1.9	1.7	0.6	1.6		0.8	0.8	0.9
4OO8_A	1.8	1.9	1.7	0.7	1.6	0.8		0.5	0.7
4UN3_B	1.8	1.9	1.7	0.7	1.5	0.8	0.5		0.7
5F9R_B	1.8	1.9	1.7	0.8	1.6	0.9	0.7	0.7	

Table S1b | Pairwise RMSDs for the Cα atoms of HNH ββα fold‡ among different Cas9 crystal structures [mean = 1.4 (0.7) Å]

PDB code	apo-Cas9			Cas9-sgRNA			Cas9-sgRNA-DNA		
	4CMP_A	4CMP_B	4CMQ_A	4ZT0_A	4ZT0_C	4ZT9_A	4OO8_A	4UN3_B	5F9R_B
4CMP_A		0.3	0.3	2.1	2.2	2.0	2.1	2.0	2.0
4CMP_B	0.3		0.4	2.2	2.3	2.1	2.2	2.1	2.1
4CMQ_A	0.3	0.4		2.1	2.1	1.9	2.0	2.0	2.0
4ZT0_A	2.1	2.2	2.1		0.7	0.3	1.0	1.0	1.1
4ZT0_C	2.2	2.3	2.1	0.7		0.8	0.7	0.8	0.8
4ZT9_A	2.0	2.1	1.9	0.3	0.8		1.0	1.0	1.0
4OO8_A	2.1	2.2	2.0	1.0	0.7	1.0		0.4	0.5
4UN3_B	2.0	2.1	2.0	1.0	0.8	1.0	0.4		0.4
5F9R_B	2.0	2.1	2.0	1.1	0.8	1.0	0.5	0.4	

†Residues 781 to 905; ‡Residues 837 to 867; *Chain identifier present in the PDB file.

Table S2 | Average pairwise Cα RMSDs of tMD-derived and cMD^{ens}-derived catalytic Cas9 aggregates relative to the crystal structure (upper) and between the two structural ensembles (lower) [Å]*

	Relative to the crystal structure [PDB code: 5F9R]¶							
	ALL†	RuvC‡	Topo	CTD	HNH	REC1	REC2	REC3
**tMD-derived catalytic state**	5.6 (0.2)	3.0 (0.2)	3.8 (0.6)	7.2 (0.7)	10.6 (0.3)	4.0 (0.4)	7.0 (0.4)	3.4 (0.3)
**cMD^{ens}-derived catalytic state**	5.7 (0.2)	2.7 (0.3)	3.0 (0.5)	6.8 (0.8)	11.5 (0.6)	3.2 (0.5)	7.9 (0.7)	2.9 (0.3)
	**Relative to the crystal structure [PDB code: 5F9R]§**							
	**ALL†**	**RuvC‡**	**Topo**	**CTD**	**HNH**	**REC1**	**REC2**	**REC3**
**tMD-derived catalytic state**	5.6 (0.2)	1.7 (0.1)	2.6 (0.5)	3.6 (0.5)	1.3 (0.2)	1.6 (0.1)	1.9 (0.1)	2.5 (0.1)
**cMD^{ens}-derived catalytic state**	5.7 (0.2)	1.7 (0.2)	2.3 (0.3)	3.5 (0.4)	1.4 (0.3)	1.5 (0.1)	1.9 (0.2)	2.2 (0.2)

Between the two differently derived catalytic states¶							
ALL	RuvC	Topo	CTD	HNH	REC1	REC2	REC3
2.6 (0.1)	1.8 (0.2)	1.8 (0.4)	3.6 (0.3)	2.5 (0.5)	2.0 (0.2)	2.0 (0.3)	2.4 (0.1)
**Between the two differently derived catalytic states§**							
2.6 (0.1)	1.2 (0.1)	1.0 (0.2)	3.1 (0.1)	1.2 (0.4)	1.2 (0.1)	1.1 (0.2)	1.9 (0.1)

* tMD, targeted MD; cMD^{ens}, ensemble conventional MD. See **Table 1** in main text.
An aggregate of 50 most populated structures were extracted for calculations based on cluster analysis (Supplementary Text)

† The whole protein

‡ Residues 1047-1071 and 1016-1031 excluded. Due to the absence of 5'-end ntDNA[5], this local binding groove exhibits remarkable opening and closing mobility.

¶ Best-fit to the Cα atoms of the whole reference protein prior to RMSD calculations

§ Best-fit to the Cα atoms of individual protein domains prior to RMSD calculations

Table S3 | Summary of the interacting pairs between Cas9 HNH domain and other components in the complex system from biased (tMD) and unbiased ensemble (cMD^{ens}) simulations and comparison with the starting pre-catalytic structure

Cas9 domain		HNH domain[*]	Interaction pattern[§]	Catalytic state [tMD][†] (occurrence %)	Pre-catalytic state [5F9R][‡]	Catalytic state [cMD^{ens}][¶]	Suggested substitution[#]
REC3	Glu584<br>Asp585	Lys775	Salt bridge/H-bond	19<br>27	-<br>-	√<br>-	
	Asp585	Arg778	Salt bridge/H-bond	16	-	√	
	Lys558<br>**Arg586**	**Glu779**	Salt bridge/H-bond	17<br>48	-<br>-	√<br>√	Arg586Ala<br>Glu779Ala
REC2	Asp261<br>Lys263	Gln805	H-bond	7<br>16	-<br>-	√<br>√	
	Lys234<br>Asn235	Asp829	Salt bridge/H-bond<br>H-bond	15<br>13	-<br>-	√<br>√	
	**Glu223**	Arg832<br>**Arg859**	Salt bridge/H-bond<br>Salt bridge/H-bond	91<br>18	-<br>-	√<br>√	Glu223Ala<br>Arg859Ala
	Thr249<br>Asn251	Asn831	H-bond	27<br>46	-<br>-	√<br>√	
	Gly247<br>Thr249	Ser834	H-bond	44<br>16	-<br>-	√<br>√	
	Ser217<br>Lys218<br>Ser219	**Asp835**	H-bond	43<br>55<br>99	-<br>-<br>-	√<br>√<br>√	Asp835Ala
BH	Thr58	Lys848	H-bond	57	-	√	
	Glu60	Lys848<br>Gln844	Salt bridge/H-bond<br>H-bond	51<br>20	-<br>-	√<br>√	
REC1	**Glu370**<br>**Glu396**	Lys862	Salt bridge/H-bond	61<br>68	-<br>-	√<br>√	Glu370Ala<br>Glu396Ala<br>Lys866Ala
	**Glu370**<br>Glu371	**Lys866**	Salt bridge/H-bond	25<br>18	-<br>-	√<br>√	
**tDNA**	DT23<br>DA24	**Arg765**	Salt bridge/H-bond	100<br>16	√<br>√	√<br>√	Arg765Ala
	DT25	**Asn767**	H-bond	92	√	√	Asn767Ala
	DT16	Lys810	Salt bridge/H-bond	45	-	√	
	DG13	**Ser845**	H-bond	93	-	√	Ser845Asp
**sgRNA**	RG2	**Arg765**	Salt bridge/H-bond	99	-	√	Arg765Ala
	RA9	**Arg780**<br>**Arg783**	Salt bridge/H-bond	100<br>72	√<br>-	√<br>√	Arg780Ala<br>Arg783Ala
	RA8	**Asn803**<br>Gln807	H-bond	94<br>37	-<br>-	√<br>√	Asn803Ala
	RA9	Tyr812	H-bond	97	-	√	Tyr812Ala
	RA12<br>RU13	Lys848	Salt bridge/H-bond	89<br>81	-<br>-	√	
	RG11	**Arg895**	Salt bridge/H-bond	99	-	√	Arg895Ala

The residues whose alanine substitution was experimentally shown to enhance Cas9 specificity are highlighted in blue (see **Supplementary Fig. 1c**). The promising candidate residues for further testing, determined based on our study, are in red boldface.

[*] Part of HNH domain flanking link regions (L1&L2) included into statistics

§ Salt bridge interaction is defined as the distance between the nitrogen and oxygen atoms is less than 4 Å; A hydrogen bond (H-bond) is defined as the distance between the donor and receptor atoms is less than 3.5 Å and the angle formed by the donor, hydrogen and acceptor atoms is less than 35° from 180°.

† Post targeted MD (tMD)-derived interactions (**G6** in **Table 1**).

‡ Presence (√) or not (-) in the initial pre-catalytic crystal structure (PDB code: 5F9R)

¶ Presence (√) or not (-) in the ensemble conventional MD (cMD^{ens})-derived catalytic state

Suggested amino acid mutations for further specificity improvement

III. Supplementary Computational Procedures

Principal Component Analysis (PCA)

PCA is a technique for transforming a series of potentially coordinated observations into a set of orthogonal vectors called principal components (PCs) and is widely used to characterize the dominant modes of motion underlying protein dynamics[6,7]. The calculations of PCs involve two main steps, i) the calculation of covariance matrix, and ii) the diagonalization of this matrix. With the goal of comparing the conformational dynamics of HNH domain between different MD simulations, the whole simulation trajectories (**G1-G4** and **G10**, **Table 1**) were first combined and superimposed to the starting crystal structure using the Cas9 Cα atoms excluding those on the HNH domain. After that, the PCA calculations were performed only on the HNH domain to determine the eigenvectors and associated eigenvalues (referred to collectively as eigenmode). The eigenvector with the largest eigenvalue corresponds to the lowest mode of motion. The PC analysis was done with the *ccptraj* module included within the AmberTools16[8].

HNH Active State Modeling & HNH Pairwise RMSD Computation

Starting from the pre-catalytic Cas9 structure (PDB code: 5F9R[5]), the detailed procedure modeling its putative catalytic state of HNH domain from the homologous T4 Endonuclease VII (Endo VII) complexed with a DNA Holliday junction (PDB code: 2QNC[9]) was illustrated in **Supplementary Fig. 5**. It should be mentioned that 2QNC represents a catalytically active state where one Mg^{2+} was coordinated at the interface between the enzyme ββα motif and scissile phosphate (see also **Figure 2c-d** in the main text), making it the best candidate for active state modeling among the available ββα-metal nuclease structures.

We took three steps to model the HNH active state. In **step 1**, the scissile phosphate and flanking nucleotides in the T4 Endo VII system (2QNC) was aligned to the corresponding tDNA stretch in the Cas9 complex of the pre-catalytic state (5F9R). In **step 2**, Cas9 HNH domain was moved toward the tDNA with the transformation matrix calculated from the paired ββα motifs in the two nucleases, resulting in a model of the HNH domain docked at the cleavage site. Notably, the equivalent residues between the above ββα motifs for transformation matrix calculation were determined based on topology-independent structure superposition by the CLICK algorithm[4] instead of generally used sequence alignment. The backbone RMSD of HNH domain between the pre-catalytic Cas9 state (5F9R) and the modeled "active" state is 25 Å (**Supplementary Fig. 5f**). In **step 3**, we repeated step 1 and step 2, replacing the crystal structure (5F9R) with snapshot structures from the sets of long cMD trajectories (**G1** and **G2**, **Table 1**). We thereby obtained a modeled "active" state for every snapshot of the simulations. We calculated RMSD between the snapshot structure and its corresponding "active" state and used it as a metric to evaluate how close the snapshot conformation to its putative active state.

Details of Generating tMD-derived Catalytic State

We employed the targeted molecular dynamics (tMD) method to drive Cas9 conformational transition. The target structures for tMD were built by reference to the catalytically active T4 Endo VII system above (**Supplementary Fig. 5**). To minimize the potential artificial effect by tMD, we extracted two snapshots from the sets of long cMD trajectories (**G1** and **G2**, **Table 1**) as the starting structures that show most proximity to the their respective modeled "active" states in terms of HNH domain conformation (**Supplementary Fig. 5**). The backbone RMSD differences for the HNH domain from the target structures are about 10 Å, which are remarkably reduced as compared with that of 25 Å if using the pre-catalytic crystal structure (5F9R) as the starting point. Accordingly, the tMD stating points were much closer to the corresponding end points in the subspace defined by the first two principal components with regard to the crystal structure (**Figure 1d**). Not surprisingly, simple docking of the HNH domain toward the putative catalytic state inevitably brings about numerous steric clashes with the other components in the complex system (**Supplementary Fig. 5f**), indicating considerable conformational rearrangements in Cas9 must be implicated during the pre-catalytic to catalytic state transition. We note that we did not employ the trajectory snapshots from aMD, albeit further approach to the target conformations with a minimum RMSD difference of ~5 Å, as it appears that the enhanced sampling via aMD also accompanies an appreciable distortion regarding the internal conformation of HNH domain (**Supplementary Fig. 4 and Supplementary Table 1**). During tMD, the Cα atoms of the protein residues (excluding HNH domain) exhibiting low fluctuations were weakly restrained with a force constant of 0.1 kcal/mol/$Å^2$ to prevent solute global drift. The guiding forces were exerted only on the HNH domain backbone atoms with a force constant of 0.5 kcal/mol/$Å^2$, and the simulation time was set to 100 ns (**G5**, **Table 1**), representing a RMSD deceasing rate of ca. 0.1 Å/ns.

At the end of tMD, the RMSD between the initial and target coordinates declined to ~0.8 Å, indicating completion of the anticipated conformation change. We selected two structure snapshots that are at near the end of tMD for subsequent cMD (**G6**, **Table 1**), in which one Mg^{2+} was introduced at the interface between the HNH domain and tDNA in the framework of the one-metal-ion mechanism (**Figure 2d**)[10,11]. Here, we did not employ the tMD end structures (i.e. at 100 ns) as the start points for Mg^{2+} introduction, given that the modeled target coordinates used in tMD do not necessarily represent a true catalytic state, and importantly, that the Mg^{2+} may assist further conformation change to bridge the distance gap for catalysis as we previously demonstrated with the RuvC domain[12]. This consideration allowed for spontaneous adaptation of the system to the catalytic conformation. The deliberate building procedures could ensure least perturbation on the system and hence eliminate potential artificial effects by the tMD that is readily subjected to question. After sufficient equilibration, we finally obtained a reasonable catalytic

conformation, featuring stable Mg^{2+}-involved coordination configuration (**Figure 2a**) that matches well with that observed in the T4 Endo VII system (**Figure 2c**).

Details of Generating cMD^{ens}-derived Catalytic State

The above tMD-based strategy to capture the catalytic state in essence is dependent on a modeled putative "target" state. One may question the reliability of the derived state and associated results, though the model was treated with careful considerations. To eliminate these concerns, we developed an ensemble sampling-based scheme targeting the active state forward. The basic idea is as follows: **i**) pre-define an a priori metric (or multiple if necessary) like distance, angle and RMSD; **ii**) use this metric to track conformational transition and screen a structure most approximate to expected target state; **iii**) perform ensemble conventional MD simulations (cMD^{ens}) starting from the above extracted structure; **iv**) screen another closest structure snapshot from previous cMD^{ens} and initiate a new cycle of ensemble simulations. Ideally, we could get closer to or even hit the target conformation through several or more cycles, depending on the energetic barrier height between the initial and target states and the sampling length accessible to each independent run.

Here we used the geometric mean of the distances of +4**P** (the scissile phosphate) to the two active residues His840 and Asp861 ($\sqrt{d_{+4\mathbf{P}-\mathrm{H840}} * d_{+4\mathbf{P}-\mathrm{D861}}}$) as a metric to monitor the HNH domain conformational change: the smaller this value, the closer to the target active state (**Figure 4a,b**). From the sets of long cMD trajectories (**G1** and **G2**, **Table 1**), we extracted a structure bearing a minimum value of ~9 Å as the starting point for ensemble simulations (**Figure 3a**), where one Mg^{2+} was placed around the reaction center as done for the post tMD simulations (see **Materials and Methods**). In each cycle, a total of 10 independent runs were carried out and each run lasted 500 ns (**G8.1-G8.4**, **Table 1**). Through four cycles, the above geometric mean got sable at ~6 Å (**Figure 3a**), which is comparable to that observed for the tMD-derived catalytic state (**Figure 4a**). Accordingly, the RMSD of the reaction interface from the tMD-derived catalytic state declined from initial ~3 Å to ~1 Å (**Figure 4b**). Moreover, the Mg^{2+}-involved coordination composition and configuration here (**Figure 2b**) are essentially the same to those derived from tMD (**Figure 2a**), except that Tyr823 was engaged to Asp839 via an intercalated water molecule, again confirming the structural role of Tyr823 around the reaction center. These observations thus demonstrated formation of the cMD^{ens}-derived catalytic state.

Cluster Analysis

The simulation structures used for visualization and comparison were determined through the cluster analysis with the package VMD (version 1.9.2)[13]. Following our previous experience with the same system[12], the reaction interface atoms were selected for calculations, involving the heavy atoms of the three

active residues, Asp839, His840 and Asp861, the Cα atoms of the remaining residues on the HNH ββα motif, the backbone of the scissile stretch on the tDNA (+3**P** to +5**P**), and the coordinated Mg^{2+} between them. By varying the RMSD cutoff (0.6~1.0 Å here), we finally obtained four groups in which the first two account for > 80% of total population. The structure(s) closest to the centroid of the largest ensemble were extracted for analysis.

Binding Free Energy Calculation & Per-residue Energy Decomposition

The end-point Molecular Mechanics-Generalized Born Surface Area (MM-GBSA) approach[14] was employed to estimate per-residue energetic contribution to Mg^{2+} binding and the difference in the affinities of the tDNA to Cas9 with and without Mg^{2+} bound at the reaction interface. Compared to the alternative Molecular Mechanics-Poisson Boltzmann Surface Area (MM-PBSA), MM-GBSA is computationally more efficient and has shown to give comparable or even better accuracy[14,15]. All the MM-GBSA calculations were performed with the program *MMPBSA.py* in AmberTools16[16]. The entropic contribution was not taken into account here, due to the high computational cost and potential convergence problem. However, omission of this term does not qualitatively affect the results[14,15]. The last 400 ns of each set of simulation trajectories were used for calculations, with 50-ps intervals. Specifically, in the case of Mg^{2+} binding free energy calculation, the three water molecules closet to the coordinated Mg^{2+} in each trajectory snapshot were retained and considered as part of the Cas9-sgRNA/tDNA "receptor".

Non-bonded Interaction Energy Calculation

The non-bonded interaction energies of the HNH ββα motif with the scissile phosphate and flanking nucleotides (+3**P** to +5**P**) were calculated by the software NAMD (version 2.12)[17], employing the same structural ensemble as mentioned above. The truncation cutoff was set to 10 Å, consistent with that used in MD simulations.

VI. Supplementary References

1 Dagdas, Y. S., Chen, J. S., Sternberg, S. H., Doudna, J. A. & Yildiz, A. A Conformational Checkpoint Between DNA Binding And Cleavage By CRISPR-Cas9. *Sci. Adv.* **3,** eaao0027 (2017).

2 Osuka, S. *et al.* Real-time observation of flexible domain movements in Cas9. *bioRxiv***,** 122069 (2017).

3 Sternberg, S. H., LaFrance, B., Kaplan, M. & Doudna, J. A. Conformational control of DNA target cleavage by CRISPR-Cas9. *Nature* **527,** 110-113 (2015).

4 Nguyen, M., Tan, K. P. & Madhusudhan, M. S. CLICK—topology-independent comparison of biomolecular 3D structures. *Nucleic Acids Res.* **39,** W24-W28 (2011).

5 Jiang, F. G. *et al.* Structures of a CRISPR-Cas9 R-loop complex primed for DNA cleavage. *Science* **351,** 867-871 (2016).

6 David, C. C. & Jacobs, D. J. Principal component analysis: a method for determining the essential dynamics of proteins. *Methods Mol. Biol.* **1084,** 193-226 (2014).

7 Amadei, A., Linssen, A. B. & Berendsen, H. J. Essential dynamics of proteins. *Proteins* **17,** 412-425 (1993).

8 Salomon-Ferrer, R., Case, D. A. & Walker, R. C. An overview of the Amber biomolecular simulation package. *Wiley Interdiscip. Rev. Comput. Mol. Sci.* **3,** 198-210 (2013).

9 Biertumpfel, C., Yang, W. & Suck, D. Crystal structure of T4 endonuclease VII resolving a Holliday junction. *Nature* **449,** 616-U614 (2007).

10 Yang, W. Nucleases: diversity of structure, function and mechanism. *Q. Rev. Biophys.* **44,** 1-93 (2011).

11 Yang, W. An equivalent metal ion in one- and two-metal-ion catalysis. *Nat. Struct. Mol. Biol.* **15,** 1228-1231 (2008).

12 Zuo, Z. & Liu, J. Cas9-catalyzed DNA Cleavage Generates Staggered Ends: Evidence from Molecular Dynamics Simulations. *Sci. Rep.* **5** (2016).

13 Humphrey, W., Dalke, A. & Schulten, K. VMD: Visual molecular dynamics. *J. Mol. Graph. Model.* **14,** 33-38 (1996).

14 Hou, T. J., Wang, J. M., Li, Y. Y. & Wang, W. Assessing the Performance of the MM/PBSA and MM/GBSA Methods. 1. The Accuracy of Binding Free Energy Calculations Based on Molecular Dynamics Simulations. *J. Chem. Inf. Model.* **51,** 69-82 (2011).

15 Zuo, Z. C., Weng, J. W. & Wang, W. N. Insights into the Inhibitory Mechanism of D13-9001 to the Multidrug Transporter AcrB through Molecular Dynamics Simulations. *J. Phys. Chem. B* **120,** 2145-2154 (2016).

16 Miller, B. R. *et al.* MMPBSA.py: An Efficient Program for End-State Free Energy Calculations. *J. Chem. Theory Comput.* **8,** 3314-3321 (2012).

17 Phillips, J. C. *et al.* Scalable molecular dynamics with NAMD. *J. Comput. Chem.* **26,** 1781-1802 (2005).
